# Molecular and *In Vivo* Characterization of Cancer-Propagating Cells Derived from MYCN-Dependent Medulloblastoma

**DOI:** 10.1371/journal.pone.0119834

**Published:** 2015-03-18

**Authors:** Zai Ahmad, Lukasz Jasnos, Veronica Gil, Louise Howell, Albert Hallsworth, Kevin Petrie, Tomoyuki Sawado, Louis Chesler

**Affiliations:** 1 Division of Clinical Studies, The Institute of Cancer Research, London, SM2 5NG, United Kingdom; 2 Division of Molecular Pathology, The Institute of Cancer Research, London, SM2 5NG, United Kingdom; University of Navarra, SPAIN

## Abstract

Medulloblastoma (MB) is the most common malignant pediatric brain tumor. While the pathways that are deregulated in MB remain to be fully characterized, amplification and/or overexpression of the *MYCN* gene, which is has a critical role in cerebellar development as a regulator of neural progenitor cell fate, has been identified in several MB subgroups. Phenotypically, aberrant expression of MYCN is associated with the large-cell/anaplastic MB variant, which accounts for 5-15% of cases and is associated with aggressive disease and poor clinical outcome. To better understand the role of MYCN in MB *in vitro* and *in vivo* and to aid the development of MYCN-targeted therapeutics we established tumor-derived neurosphere cell lines from the GTML (*Glt1-tTA/TRE-MYCN-Luc*) genetically engineered mouse model. A fraction of GTML neurospheres were found to be growth factor independent, expressed CD133 (a marker of neural stem cells), failed to differentiate upon MYCN withdrawal and were highly tumorigenic when orthotopically implanted into the cerebellum. Principal component analyzes using single cell RNA assay data suggested that the clinical candidate aurora-A kinase inhibitor MLN8237 converts GTML neurospheres to resemble non-MYCN expressors. Correlating with this, MLN8237 significantly extended the survival of mice bearing GTML MB allografts. In summary, our results demonstrate that MYCN plays a critical role in expansion and survival of aggressive MB-propagating cells, and establish GTML neurospheres as an important resource for the development of novel therapeutic strategies.

## Introduction

In children, medulloblastoma (MB) is the most frequently occurring primitive neuroectodermal tumor (PNET) originating in the brain and has been classified into four major subtypes based on copy number variation and transcription profiles: MB^WNT^, MB^SHH^ (sonic hedgehog), MB^Group3^ and MB^Group4^ [1,2]. Despite the advances made in MB molecular classification, in the majority of pediatric MB (with the exception of aberrant SHH and WNT pathways) oncogenic signaling pathways that are therapeutically targetable remain to be identified. Recent research has, however, demonstrated that MB harboring MYCN amplification and/or overexpression may also be targeted [3]. Amplification of the *MYCN* gene is associated with a poor prognosis [4,5], is observed in MB^SHH^, MB^Group3^ and MB^Group4^ subtypes [6–10] and is aberrantly expressed in the majority of human MB [11]. Despite the growing importance of MYCN as a therapeutic target in MB, however, we still have a poor understanding of how aberrant *MYCN* expression transforms neural stem/progenitor cells to tumors. We previously reported a genetically engineered mouse model (GEMM) of MYCN-driven MB (GTML: *G* *lt1-tTA/* *T* *RE-* *M* *YCN-* *L* *uc*) in which MYCN expression is controlled by doxycycline (dox) (Tet-off). Using this system we showed that targeted expression of MYCN induces both classic and large cell anaplastic (LCA) pathology independent of SHH [11]. Subgroup classification based on gene expression profiles derived from human medulloblastoma samples in comparison to transgenic mice suggested that GTML and GTML/Trp53^KI/KI^ mice displayed expression profiles characteristic of human MB^Group3^ [3]. Neurosphere lines established from GTML mice proliferate robustly, express neuronal markers, and form tumors when orthotopically implanted into the brains of mice [12]. These results suggest that GTML neurosphere cultures contain tumor-propagating cells, making this a potentially useful system with which the transformative role of MYCN in MB genesis could be elucidated.

In this context, we conducted a series of molecular and cytological studies to characterize neurosphere cultures established from GTML mice. Single cell analysis revealed the expansion of a homogeneous cell population is dependent on MYCN expression for survival. These spheroids are resistant to differentiation, and have features characteristic of partially-committed neural stem and progenitor cells with MYCN expression, including upregulation of Nestin, CD133, Otx2, and Musashi, expression of which are associated with maintenance of an undifferentiated state, and typical of MB-stem/progenitors. Cerebellar implantation of GTML neurospheres subpopulations showed that tumor propagating-potential resided in CD133+ but not CD15+ or CD15- cells. Furthermore, treatment of tumors with MLN8237, an aurora-A kinase inhibitor that induces MYCN oncoprotein degradation [13], effectively extended the survival rate of GTML mice with aggressive MB. These results clarify the role of MYCN in cellular transformation and progression of MB, strengthening the hypothesis that MYCN drives the expansion and enrichment of CD133+ tumor-propagating cells capable of generating aggressive MB.

## Materials and Methods

### Mouse husbandry

The GTML mouse model has been described previously [11]. All mouse procedures were approved by the Institute of Cancer Research Ethical Committee following UK Home Office guidelines (Project License Number: 70/7945). All surgery was performed under anesthetic isoflurothane, and all efforts were made to minimize suffering.

### Neurosphere isolation and culture

Tissue was isolated from GTML tumors or postnatal P5, P8, P21 cerebellum and midbrain and transferred into cold HBSS (Invitrogen). The tissues were then cut into 2–3mm^2^ pieces and enzymatically dissociated at 37°C using Liberase Blendzyme 1 (0.62 Wunsch units/ml, Roche) in PBS for 15–45 min. The enzyme reaction was stopped using 10% volume of Fetal Calf Serum (FCS, PAA) before the cells were triturated in DMEM/F12 media containing B27 and filtered through a 70μm mesh. Subsequently the cells were cultured in DMEM/F12 medium (Invitrogen) supplemented with 2% B27 supplement (Invitrogen), 20ng/ml epidermal growth factor (EGF, Sigma), 20ng/ml fibroblast growth factor (bFGF-basic, Invitrogen) and 100 units/ml penicillin/streptomycin.

To examine cell division rates, neurospheres were dissociated by pipetting, and cells stained with trypan blue were counted using a hemocytometer. Alternatively, cells were counted using the Guava PCA-96 instrument after staining with Guava ViaCount Flex reagent (1:50, Millipore), according to the manufacturers protocol.

For assessment of differentiation potentials, cells were plated onto coverslips coated by poly-D-lysine (0.1M, Sigma), 0.1% gelatin or laminin (0.5μg–2.0μg/ml, Invitrogen) and grown under differentiation conditions for up to 14 days. Differentiation medium is composed of DMEM/F12 medium supplemented with 10% FCS, B27 with or without 1 μg/ml retinoic acid (Sigma Aldrich). We also plated freshly sorted GTML cells in the presence of 0.5–1.0mg/ml of collagen (Invitrogen; A1048301) in the pro-differentiation neurobasal media with serum.

### Orthotopic implantation

To examine the tumorigenic potential, cells directly obtained from primary tumors, GTML spheres, or their derivatives were resuspended in neurosphere media and injected into the cerebellum of FVB/N female mice: following numbers of cells were implanted: primary tumors (25 or 100 cells/site), M10519 cells (passages 10–27, 25 to 10,000 cells/site) or freshly sorted cells (10 cells/site).

Mice aged 6–8 weeks were anesthetized using inhalation of anesthetic isoflurothane. An incision (1cm^2^) was made in the midline of the scalp over the cerebellum, and a small hole with a diameter of 1mm was made in the skull using a dental drill at a position 1mm lateral from the midline and between 1 and 2 mm posterior to the lambdoidal suture avoiding any obvious blood vessels. Cells were injected at a depth of 2 mm using a 10 μl Hamilton syringe in a stereotaxic frame. The needle was left in place for a further 2 minutes to avoid reflux. The needle was removed gently to avoid dislodging tumor cells. The skull was then sealed using surgical glue. After the implantation tumor progression was monitored weekly using the IVIS Living Image System (Caliper Life Sciences) according to the manufacturer’s instructions. Luciferase imaging was conducted as described previously [11] except that D-luciferin potassium salt (Parkin Elmer) in PBS was used at 15 mg/kg.

Doxycycline treatment was conducted as described in our previous paper [11]. Briefly, mice with progressed tumors measured with a signal of 1x10^9^ photons/s were fed with chow (TestDiet) containing doxycycline at 200 mg/kg to provide a daily dose of approximately 32 mg/kg. Tumor burden was determined by bioluminescence.

### Immunohistochemistry and Immunocytochemistry

For immunocytochemistry neurospheres were embedded in OCT mounting media (BDH) and slowly frozen using isopropanol cooled in liquid nitrogen. Then 4μm thick cryosections were mounted on poly-lysine coated slides (VWR) and fixed with 4% paraformaldehyde (PFA, Sigma) in PBS for 10 minutes. After subsequent washes with TBS the sections were then blocked with 10% bovine serum albumin (BSA, Sigma), 15mM glycine (Sigma) and 0.02% Triton x100 (Sigma) in TBS for 1 hour at room temperature. For mouse antibodies sections were blocked using the MOM blocking kit (Vector Laboratories) according to the manufacturer’s instruction. Sections were incubated with primary antibody overnight in blocking solution (0.1%BSA-PBS) at room temperature and after subsequent washes with TBS slides were labelled with secondary antibodies conjugated with Alexa Fluor (1:500, Molecular Probes). Slides were counterstained with DAPI (Sigma) and then were mounted with Fluoromount G (Southern Biotech).

For immunohistochemistry, tumor samples were fixed in 4% paraformaldehyde in PBS for 24hrs to 7days. Samples were decalcified with 0.3M EDTA and then processed using the Leica ASP300S tissue processor to create a paraffin wax block. Sections were cut at 4μM for hematoxylin and eosin staining and immunohistochemistry was conducted as previously described [14].

Antibodies used for immunohistochemistry and immunocytochemistry were: MYCN (OP-13, Calbiochem), Ki67 (556003, BD Pharmingen), GFAP (Z0334, DAKO), CD15 (332778, BD Bioscience), Tuj1 (BAM1195, R&D), Cleaved Caspase 3 (9664, Cell Signaling).

### Immunoblot analysis

Neurospheres were cultured under normal and differentiation conditions in the presence or absence of 1μg/ml doxycycline. Spheres were harvested, and then suspended in cell lysis buffer (Cell Signaling Technology) according to manufacturer’s protocol. Immunoblot analysis was performed as described [14]. Following antibodies were used: MYCN (OP-13, Calbiochem), c-MYC (SC-40, Santa Cruz), Nestin (ab5968, Abcam), beta-actin (4967, Cell Signaling Technology) and GAPDH (2118, Cell Signaling Technology).

### Flow cytometry and fluorescence-activated cell sorting

For cell cycle analyses, GTML spheres were treated with 1μg/ml doxycycline and samples were taken at 0, 2, 4, 6, 8, 24, and 48 hours. The samples were fixed using 70% ice-cold ethanol, and then were stored at 4°C for at least 30 min. Then cells were stained with propidium iodide (PI) (40μg/ml, Invitrogen) at 37°C for 30 min, and then were stored at 4°C for 24 hours. Cell cycle profiles were analyzed using a Becton Dickinson LSR II fluorescence-activated cell analyzer.

To isolate the CD15+ and CD15- or CD133+ and CD133- populations, M10519 GTML neurospheres were dissociated into single cells, and then were stained with anti-CD15 conjugated with FITC (1:100, Millipore) or anti-CD133 conjugated with PE (1:100, Miltenyi Biotec) for at 30 min on ice, washed with PBS containing 0.3% Bovine Serum Albumin with antibiotics. Cells were then counterstained with 0.5μg/ml of DAPI in PBS and sorted through a Becton Dickinson FACS Aria. Cells were sorted into DMEM/F12 media containing growth factors and B27. The purity of each population was assessed at the end of each sort using the same analyzer.

### Purification of CD133+/- cells using magnetic beads

M10519 GTML neurospheres were dissociated into single cells using the Neurosphere Dissociation kit P (Miltenyi Biotec) following manufacturer’s instruction. After the dissociation, cells were centrifuged at 500xg for 10min and then pellet was resuspended in 1ml of PBS supplemented with 2%FBS and 1 mM EDTA (sorting buffer). Isolation of CD133+ and CD133- populations was initially performed through magnetic separation (Easysep, Stemcell Technologies), followed by a final purification step using the FACS ARIA (BD Biosciences). Cell suspension (0.5ml, 2x10^7^ cells) was incubated with 50μl of anti-CD133 (Prominin-1) PE-conjugated antibody (Miltenyi Biotec) or anti-IgG_1_ conjugated with PE (Miltenyi Biotec) for 10 min at 4°C. Cells were then washed with sorting buffer and incubated with 100μl of PE-selection cocktail (EasySep PE selection kit, Stemcell Technologies) per 1ml of sorting buffer for 15 min at room temperature following addition of EasySep 50μl nanoparticles per 1ml of cell suspension prior to magnetic separation. To improve the purity, the magnetic separation process was repeated total three times. The purity of bead-bound or -unbound fractions was assessed using the FACS LSRII analyzer (BD Biosciences). Both CD133+ and CD133- fractions were further purified from bead-bound and—unbound fractions, respectively, using the FACS ARIA (BD Biosciences). 7AAD (Beckman Coulter) was used to exclude dead cells in FACS analyses. Live cells were counted with trypan blue staining prior to orthotopic implantation (10 cells per site).

### Limited dilution analysis

The M10519 cells (100, 10, and 1 cell(s) per 100 μl of neurobasal media in the presence of growth factors) were plated into a 96 well plate for prolonged culture. The cells were monitored bi-weekly to assess sphere formation, and then the final estimate was made approximately 4 weeks post seeding.

### Primers for single cell assays

For this study, we initially designed 48 primers for (i) factors that are expressed in three distinct MB subtypes [5,15–17], (ii) known neural stem cell markers, (iii) known cancer markers and (iv) MYCN targets. We examined these together with 93 primers we used for mouse embryonic stem cells in our previous studies [18,19] for primer validation using cDNA from GTML and mouse embryonic stem cells. Out of 141 primers, we selected 96 primers that passed the primer validation, the process of which has been described previously [18].

### Cell sorting for single cell assays

The accuracy of sorting process was determined as follows. First, 2x10^5^ cells were stained using 0.4μl Carboxyfluorescein diacetate N-succinimidyl Ester (CFSE) fluorescent dye (Sigma Aldrich), and single cells were sorted using a cell sorter Becton Dickinson into AG480F slides (Beckman Coulter). Then the presence of labeled cells was inspected under a fluorescent microscope CKX41 (Olympus) to confirm that only one cell was spotted at a reaction site of the slide. If two cells were found at a single reaction site, calibration of the sorting machine was restarted from the beginning. Once sorting accuracy was confirmed, sample cells were labeled with propidium iodide, sorted into PCR tubes, and then frozen. Cells were then thawed on ice to disrupt the cell membranes.

### Reverse transcription and target-specific amplification

The experimental detail is described in our previous paper [18]. Briefly, reverse transcription and cDNA amplification were conducted as follows. The reaction was incubated at 50°C for 15 minutes, 95°C for 2 minutes, followed by 18 cycles of 95°C for 15 second and 60°C for 4 minutes. Unincorporated primers were subsequently removed by treating with Exonuclease I (Exo I, NEB). A reaction mixture contains 10 μl of amplified cDNA, 0.4μl of 10 x Exo I buffer, 0.8μl Exo I enzyme (20u/μl), and 2.8μl of RT-PCR grade water. Reaction was held at 37°C for 30 min, then at 80°C for 15 minutes to inactivate of Exo I. Finally, DNA Suspension Buffer (36μl) was added to each sample (total 50μl).

### Analyses using BioMark system

Experimental details are described in our previous papers [19] [18], except that we used 96x96 Gene Expression IFC Array plates (Fluidigm Corporation) in this study. Sample and primer mixtures were loaded separately to inlets located on both sides of the array. A BioMark qPCR reaction mix was composed of 2.5μl of 2xTaqMan Gene Expression Master Mix (Applied Biosystems), 0.25μl of 20x DNA Binding Dye Sample Loading Reagent (Fluidigm Corporation), 0.25μl of 20x EvaGreen DNA binding dye (Biotium) and 2μl of Exo I-treated cDNA sample. A primer reaction mix was composed of 2.5μl of 2xAssay Loading Reagent, 1.25μl of DNA Suspension Buffer, and 1.25μl of a 20μM primer pair. Sample loading was done according to the instruction manual of the BioMark system.

### Data validation

To evaluate the formation of non-specific amplification products due to primer-dimer formation or non-specific annealing, we conducted melting curve analyses for each primer as previously described [18].

### Statistical analysis

Cq values higher than the threshold for reliable Cq values (Cq_threshold_, S1 Table) were omitted from the statistical analyses as described [18]. Principal component analysis and hierarchical clustering analysis (Ward’s method) were conducted using R. It should be noted that for clustering analyses and principal component analysis, Cq values higher than Cq_threshold_ were treated as unreliable values, and thus were all replaced with Cq_threshold_+1d. The Kolmogorov-Smirnov test was used to evaluate the differences of correlation coefficient distributions of data sets derived from two sample sets, due to the presence of skewness of the distribution and uneven number of observations between compared groups, both of which were not suitable for conducting stronger tests. The Log-rank test was used to evaluate the difference of the survival distributions of two sample groups.

## Results

### Establishment of GTML neurosphere lines

In this study we aimed to identify tumor-propagating cells in MB, and to establish an efficient pre-clinical study system to examine the potential of small molecules in preventing tumor development. We utilized the *Glt1-tTA/TRE-MYCN-luciferase* (GTML) transgenic mouse model, in which suppression of human MYCN and luciferase is achievable in a dox-dependent manner in brain tissue [11,12] (S1 Fig.). In this system, tumor development is preventable by dox, and tumor progression is visible using *in-vivo* bioluminescent imaging (S1 Fig.); both tumor burden and *in vitro* cell growth is linearly correlated with luciferase signal intensity (S1 Fig.) [11].

**Fig 1:**
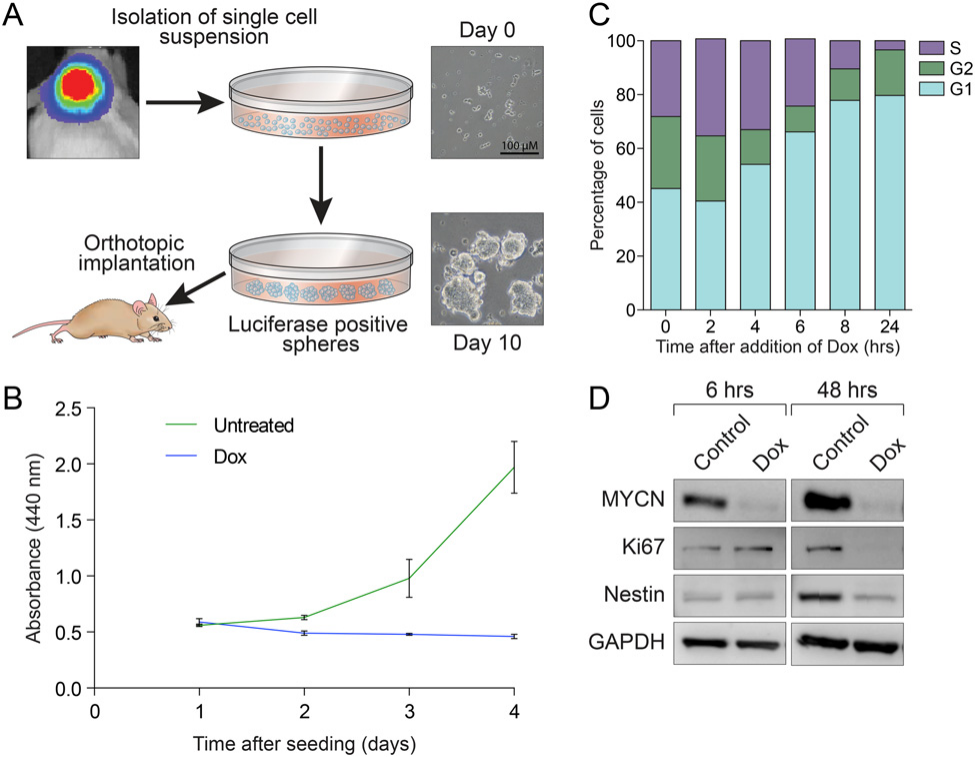
Characterization of GTML spheres. (**A**) Establishing GTML spheres in culture. Primary cells were isolated from GTML tumors, which were identified through the bioluminescence signals (upper left) and morphology, and were then dissociated into single cells. M10519 GTML neurospheres cultured after 10 days are also shown. Scale bar: 100μm. (**B**) Effect of MYCN withdrawal on cell growth. M10519 GTML spheres were seeded at density of 1x10^5^ cells/ml in the presence of absence of 1μg/ml of dox, and the proliferation of spheres were measured by WST-1 metabolic assay. Error bars, ±SD. (**C**) Effect of MYCN withdrawal on cell cycle. M10519 GTML neurosphere were treated with dox prior to cell cycle analyses using FACS. (**D**) Western analyses. M10519 GTML spheres were cultured with 1μg/ml of dox.

Primary tissues were surgically isolated from growing tumors monitored by weekly luciferase imaging (Fig. 1A and S1 Fig.). The excised tumors were dissociated and cultured in serum-free neurobasal media containing EGF and bFGF [20], and established neurospheres within 3–7 days (Fig. 1A), in contrast to explants of midbrain or cerebellum from wild type mice (which had a limited life span of 7–10 passages). Cells established from at least 6 different primary tumors at various ages were immortal and exhibited a doubling time of approximately 24 hrs. (S2 Table and S1 Fig.). Taken together these data suggest the existence of a highly proliferative, transformed cell most likely driven by MYCN transgene expression.

To examine the role of MYCN in the growth of GTML cells, we treated M10519 GTML neurospheres (as well as additional cell lines, see S2 Fig.) with dox, and found clear evidence that growth is dependent on MYCN (Fig. 1B). Cell cycle analyses using flow cytometry showed clear accumulation of growth-restricted cells in G1 phase, within 4 to 6 hours of treatment (Fig. 1C), Growth restriction was coincident with complete suppression of MYCN, but not c-Myc protein (S1 Fig. and S3 Fig.), reduced levels of Ki67, a proliferation marker, and Nestin, a neural stem and/or progenitor marker, at 48 hours after withdrawal of MYCN (Fig. 1D and S1 Fig.). Interestingly, in contrast to our previously-established GTML lines with wild type *Trp53* [12], arrested GTML cells rapidly expanded after removal of dox (S1 Fig.), suggesting that MYCN withdrawal is cytostatic in a fraction of these cells and that growth arrest is reversible. The inconsistency among the GTML cells utilized in the two studies may be due, at least in part, to the fact that all of GTML cells established in the present study harbor spontaneous mutations in the region of the *Trp53* gene encoding the p53 DNA-binding domain [3]. We undertook analysis to establish whether compensatory upregulation of mouse c-Myc protein is involved in the release from cell cycle arrest and found that c-Myc levels were constant (S1 Fig. and S3 Fig.), suggesting that at least in our neurosphere cultures, c-Myc does not compensate for the reduction of MYCN (as previously reported to occur in neural progenitors [21]). Clonogenic potentials of M10519 cells, one of GTML lines, were examined through secondary sphere assays, showing that 42% of single M10519 cells were capable of forming spheres in culture (S4 Fig).

### MYCN expression drives expansion of cells expressing markers typical of neural stem and/or progenitor cells and MB

**Fig 2:**
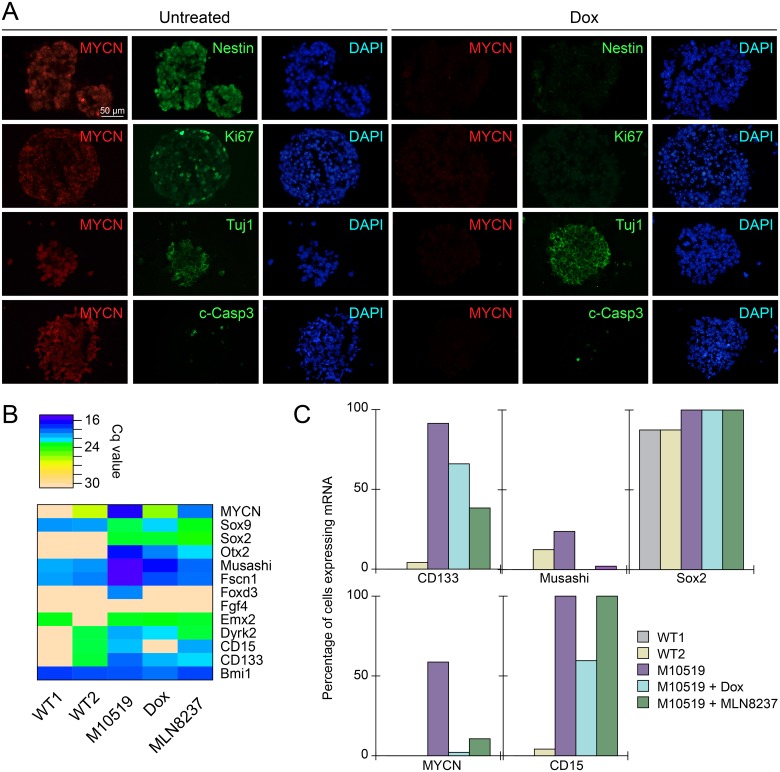
Expression of neuronal, proliferation, and/or MB markers in GTML spheres. (**A**) M10519 GTML spheres were treated with or without dox for 17 days. Immunofluorescence analysis was performed on cryosectioned spheres with anti-Ki67, anti-Nestin, or anti-Tuj1, anti-GFAP, and anti-c-Caspase 3 together with anti-MYCN. Nuclei were counterstained with DAPI. Bar, 50μm. (**B**) A heat map for averaged expression levels (Cq values, n = 96) of 13 neural stem cell genes are shown. (**C**) Percentages of cells expressing CD133, Musashi, Sox2, MYCN, and CD15 are shown. We examined WT1 and WT2 cells (see the text), untreated M10519 spheres (M10519), M10519 spheres treated with dox for 24 hours (Dox), or M10519 spheres treated with MLN8237 for 24 hours (MLN8237).

To characterize GTML neurospheres, we examined the expression of neural stem/progenitor cell markers by immunocytochemistry. We found that Nestin, a marker for neural stem/progenitor cells, and the proliferation marker Ki67 were expressed in GTML neurospheres in a MYCN-dependent manner (Fig. 2A). Expression of a neuron-specific progenitor marker Tuj1 [22] was not however visibly altered by MYCN withdrawal (Fig. 2A), implying that depletion of MYCN does not dramatically affect the degree of differentiation in culture. In contrast to our previous observation in GTML tissues [11], no prominent decrease of Cleaved Caspase 3, a marker of apoptosis, was observed upon MYCN withdrawal (Fig. 2A and S3 Fig.). To obtain a more detailed picture of gene expression at the single cell level, we analyzed a M10519 line (we found GTML lines we established in this study were remarkably similar) using 96-plexed single cell qRT-PCR (S5 Fig. and S6 Fig.). This analysis indicated that common markers for MB and neural stem cells including NeuroD1 [23], CD133 (Prominin 1) [24–27], Otx2 [11,28], and Musashi [29] were downregulated by MYCN withdrawal (Fig. 2B and S5 Fig.). The frequency of cells expressing markers including CD133 and Musashi was also decreased by dox, while other stem cell markers including Sox2 were unaffected by MYCN withdrawal (Fig. 2C and S5 Fig.). Expression of markers for astrocytes and ependymal cells (GFAP) [30], astrocytes (S100b) [31], granule neuron progenitors and glioma (Olig2) [32–34] were barely detectable (S5 Fig.) or rarely observed (S5 Fig.) in single cell analyses. Correlating with the rapid growth in culture, MYCN expression up-regulated master regulators for cell cycle progression, E2F1 and E2F2, both of which are MYCN targets [35] (S5 Fig.). CD133, a marker for S, G2, and M phases of neural stem cells [36] (Fig. 2B and 2C). MYCN, as expected, was barely detectable in M10519 cells treated with dox (Fig. 2B and 2C). Taken together, these results suggest that MYCN expression results in the expansion of cells with markers typical of neural stem cells and/or progenitors associated with MB tumorigenesis.

96-gene (see S3 Table and Janos *et al* [19]) expression profiles for 96 single M10519 cells displayed a degree of heterogeneity (S6 Fig.). Single cell BioMark HD expression data are shown in S3 Table. To evaluate the difference of correlation coefficient distributions of data sets derived from two populations, using the Kolmogorov-Smirnov test we calculated pairwise correlation coefficients of expression levels for 96 genes in a given cell population. The mean correlation coefficient (*r̄*) for M10519 neurospheres was 0.77, and the difference between M10519 cells and WT1 (*r̄* = 0.71, N_M10519_ = 1081, N_WT1_ = 990, D = 0.2662, *P* = 2.2x10^-16^) or WT2 (*r̄* = 0.60, N_M10519_ = 1081, N_WT2_ = 990 D = 0.57, *P* = 2.2x10^-16^) cells was statistically significant. These data indicate that while the expression profiles of individual cells in the M10519 neurosphere population are not entirely homogeneous in nature, the transcription profiles among cells in WT1 and WT2 neurosphere cultures derived from normal cerebella are significantly more heterogeneous. Furthermore, MYCN withdrawal resulted in a significant reduction of the heterogeneity index (*r̄* = 0.69, N_M10519_ = 1081, N_M10519+Dox_ = 990, D = 0.27, *P* = 2.2x10^-16^). Taken together, these results suggest that MYCN expression elevates the level of cellular homogeneity and expands cells with neural stem and progenitor markers.

### MYCN drives expansion of cells resistant to differentiation stimuli in culture

Interestingly, despite the stem cell and/or progenitor-like transcription profile of the expanded cells in neurosphere cultures, GTML cells expressing MYCN displayed only limited differentiation potential *in vitro*. Following 8 days of growth in serum-containing pro-differentiation media, we found that three GTML lines retained morphological features typical of undifferentiated cells (S2 Fig.), although we found that the neural stem/progenitor marker Nestin was downregulated after 2 days of MYCN withdrawal (Fig. 1D). By contrast, the same conditions induced differentiation of neurospheres established from wild type cerebella (S2 Fig.). The apparent loss of differentiation potential in GTML cells suggest that genetic or epigenetic change may occur after prolonged MYCN activation. We also found that GTML cells failed to fully differentiate under a variety of conditions (e.g. treatment with retinoic acid, a strong inducer of differentiation (S2 Fig.), or culturing in media containing 0.5mg–1mg/ml of collagen (data not shown)). When M10519 cells were grown in pro-differentiation media containing serum and retinoic acids, while we observed the downregulation of MYCN, which is controlled by the *Glt1* promoter that is activated in progenitors, and Nestin at 48–96 hours, we observe no considerable changes in the levels of neuronal markers Synaptophysin and Tuj1 and glial marker GFAP (S3 Fig.). Taken together, these results suggest that MYCN expression results in the enrichment of cells with markers typical of stem and/or progenitor cells, driving expansion of highly proliferative cells resistant to differentiation stimuli. The apparent loss of differentiation potential in GTML spheres is in contrast with neurospheres derived from human MB (for which *MYCN* status was not examined) [27] or the *Ptc*-/- mouse model [37], suggesting that persistent expression of MYCN drives irreversible commitment to expansion of cells with neural stem and progenitor markers.

### MYCN-driven GTML neurospheres retain properties of primary tumors *in vivo*

**Fig 3:**
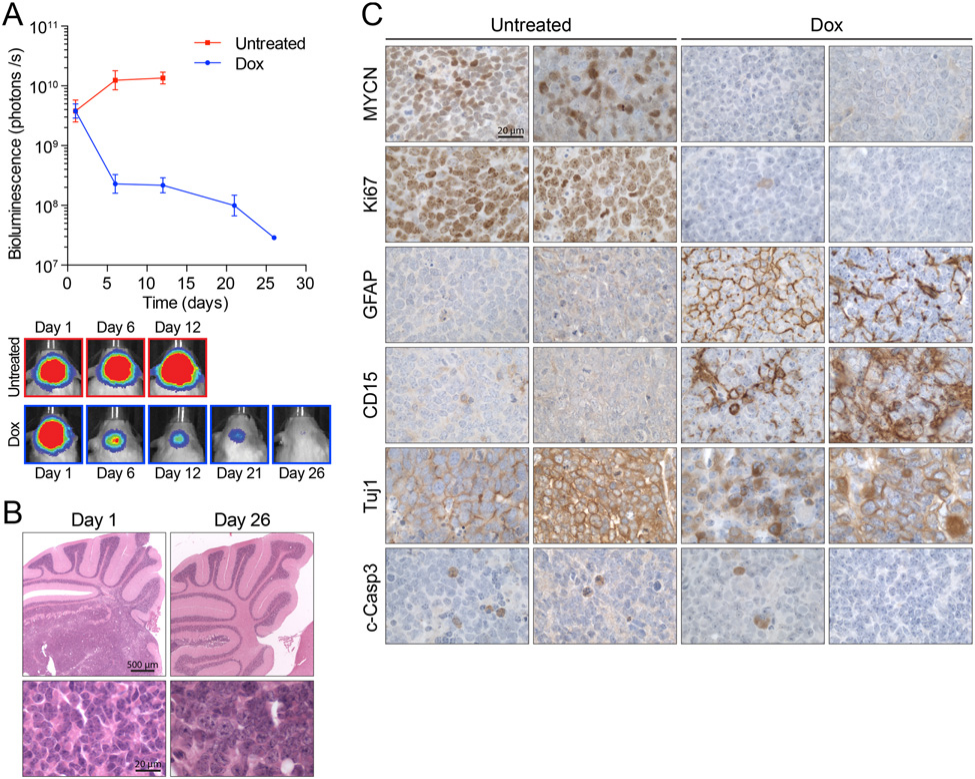
Characterization of GTML orthotopic tumors. (**A**) M10519 GTML spheres (passage 10–27) were orthotopically implanted into the cerebellum of FVB/N mice, and tumor development was monitored by bioluminescence. Once bioluminescence signal were reached at 5x10^9^ photons/s the tumors were either treated with dox to induce MYCN withdrawal or left untreated (n = 6 per treatment arm) and then the level of luciferase expression was monitored until day 26. (**B**) H&E staining of orthotopic tumors. Mice were treated with dox on day 1 (left panels) on tumors with 5x10^9^ photons/s. Tumor regression at day 26 is shown (right panels). Bars, 500μm (upper panels) and 20μm (lower panels). (**C**) Immunoperoxidase staining of paraffin-embedded orthotopic tumors. Tumors generated after the implantation of M10519 cells were harvested, paraffin-embedded, sectioned, and labeled with antibodies shown. Sections were counter-stained with hematoxylin. Bar, 20μm.

We recently showed that neurospheres derived from GTML mice are capable of forming tumors in a MYCN-dependent manner when orthotopically implanted in the cerebella of non-transgenic littermates [12]. As expected, the addition of dox to diet rapidly reduced bioluminescence (Fig. 3A) and tumor burden (Fig. 3B) in mice implanted with M10519 cells. To evaluate the impact of MYCN withdrawal *in vivo*, we examined expression of biomarkers in orthotopic tumors. Immunohistochemical analysis (Fig. 3C) showed that tumor regression induced by MYCN withdrawal correlated with the loss of expression of the Ki67 proliferation marker. While GTML cells grow as neurospheres in culture even in the presence of differentiation inducers (S2 Fig.), in a tissue environment 26 days post-initiation of dox treatment, levels of the glial marker GFAP were elevated, the staining pattern of neuronal marker Tuj1 was altered to a mosaic “salt and pepper” fashion, and MYCN displayed increased cytoplasmic localization (Fig. 3C). Interestingly, CD15 (SSEA-1/Lex), a putative marker for medulloblastoma in *Ptc1* mutant mice [38,39], also displayed elevated and distributed staining profiles upon MYCN withdrawal. We did not find the elevation of c-Caspase 3, a marker for apoptosis, by MYCN withdrawal suggesting that the tumor regression by MYCN withdrawal was caused by mechanisms other than increasing levels of cell death, with cell-cycle arrest and induction of senescence likely candidates [11]. These results suggest that in the tissue environment tumor regression by MYCN withdrawal is involved in partial differentiation and loss of proliferation, but is not associated with apoptotic cell death.

### Tumorigenic potential of GTML neurospheres resides in CD133+ cells

While the cancer stem cell hypothesis predicts that only a minority of cancer cells within a tumor are usually endowed with tumorigenic potential, our orthotopic implantation assay suggested that both primary tumor and GTML spheres comprise large number of cancer-propagating cells (S7 Fig.). Limiting dilution experiments established an 80% or 50% efficiency for tumor formation from as few as 100 implanted GTML (passages 10–27) or primary tumor cells (S7 Fig.), suggesting that MYCN acts to expand a cancer propagating cell within neurosphere cultures. We compared M10519 (passage 13) and M14942 lines (passage 11) and the difference in tumor incidence following orthotopic implantation was not statistically significant (Log-rank test, *P* = 0.4480) (S7 Fig.). Both neurosphere lines remained highly tumorigenic after 20 passages (data not shown).

To identify cancer-propagating cells enriched in neurospheres, we performed flow cytometry for CD133 and CD15, two neural stem cell surface antigens that have been identified as markers of tumor-propagating potential in MB [25,27,38,39]. Given that our single cell expression data suggested that both CD15 and CD133 were considerably upregulated by MYCN expression in M10519 GTML neurospheres (S5 Fig. and S6 Fig.), we examined whether expression of either marker was associated with tumor-forming potential. Fluorescence activated cell sorting (FACS)-based CD15 expression revealed that when MB cell populations positive or negative for this marker were orthotopically implanted, survival was not significantly altered for either the M10519 (*P* = 0.9157, Log-rank test) or M0983 (*P* = 0.8172, Log-rank test) neurosphere cell lines (S8 Fig.). The *in vivo* results correlated with *in vitro* data showing that CD15 and CD15 status had no significant effect on self-renewal and neurosphere formation *in vitro* (S8 Fig.). In contrast to CD15, FACS sorting of M10519 neurosphere cells based on CD133 expression (S9 Fig.) revealed a significant effect on the tumor-propagating potential of orthotopically implanted cells. Serial dilution revealed that mice implanted with 10 CD133+ cells developed tumors in contrast to CD133- cells, which were significantly less efficient (Log-rank test, *P* = 0.0197) (S9 Fig.). Again, these results mirrored in vitro data showing that neurosphere cells isolated on the basis of CD133 expression exhibited enhanced self renewal and ability to form neurospheres *in vitro* (S9 Fig.).

**Fig 4:**
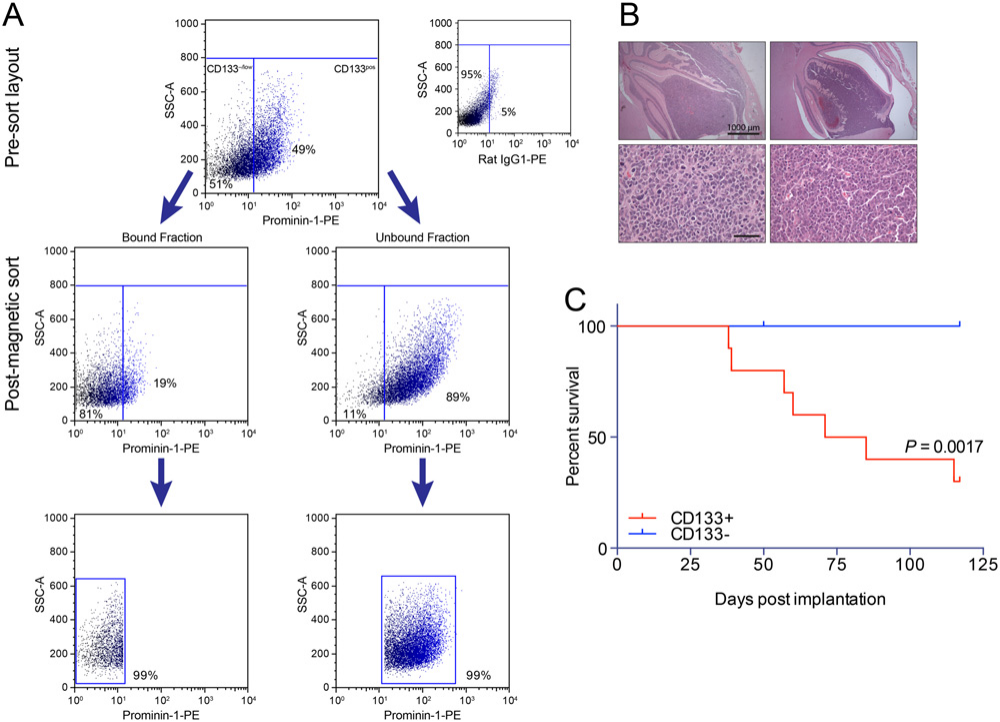
Tumor-propagating potential of CD133+ cells. (**A**) Purification of CD133+ and CD133- cells. M10519 cells (passage 13) were stained with anti-IgG1 or anti-Prominin-1 (CD133) conjugated with PE prior to purification. Stained cells were first separated into bead-bound and bead-unbound fractions by magnetic beads conjugated with anti-PE (middle panels). Then each fraction was further purified by FACS sorting. **(B**) Orthotopic tumors generated after implantation of CD133+ cells (10 cells per mouse). Sections were stained with hematoxylin and eosin. Bars, 1000μm (upper), 50μm (lower). (**C**) Kaplan-Meier curves for overall survival of mice implanted with 10 CD133+ (red, n = 10) or CD133- (blue, n = 10) M10519 cells per site.

Given that single cell expression analysis showed that the great majority of M10519 GTML cells expressed CD133 (Fig. 2C), it was possible that a small number of contaminating CD133+ cells in the CD133- fraction were responsible for residual tumor forming activity (S9 Fig.). To address this issue we sought to obtain more highly purified CD133+ and CD133- populations through anti-CD133 antibody-based magnetic separation followed by FACS (Fig. 4A). Purified CD133+ or CD133- cells were then implanted into the cerebellum of FVBN mice (10 cells per site). Mice implanted with CD133+ cells developed aggressive tumors within 50 days (n = 10) (Fig. 4B), in contrast to mice implanted with CD133- cells, which failed to develop tumors (n = 10) (Fig. 4C, *P* = 0.0017, Log-rank test). Taken together, these results suggest that MYCN acts to clonally expand highly proliferative tumor-propagating cell in GTML neurospheres resembling a stem and/or progenitor-like cell marked by CD133 positivity.

### Pharmacologic inhibition of MYCN is effective against GTML MB

Amplification of both *MYC* and *MYCN* occurs in poor-outcome MB^Group3^ and MB^Group4^ [6–10]. MYCN expression is also induced by SHH signaling and as such is highly expressed in MB^SHH^ [1,12,15,40–43]. Drugs that interfere with MYC family oncoprotein stability or function have therefore been suggested as potential therapeutic strategies to treat these MB subtypes. We therefore examined the effect of an Aurora-A kinase inhibitor MLN8237 on GTML MB. Aurora-A associates with MYCN, preventing Fbxw7-mediated proteasomal degradation of MYCN [44]. Our previous results in neuroblastoma cell lines suggested that MLN8237 blocks the interaction between Aurora A and MYCN, exposing MYCN to proteasomal degradation [13]. MLN8237 is effective in MYCN-driven transgenic mouse models of neuroblastoma (TH-MYCN), in which high-level expression of MYCN is driven in neural crest by a tyrosine hydroxylase (TH) promoter [13,45]. Recently Lehman’s group and we have independently shown that MLN8237 given to mice with tumors penetrated through the blood-brain barrier [3,46], causing the reduction of MYCN levels in brain tissues.

**Fig 5:**
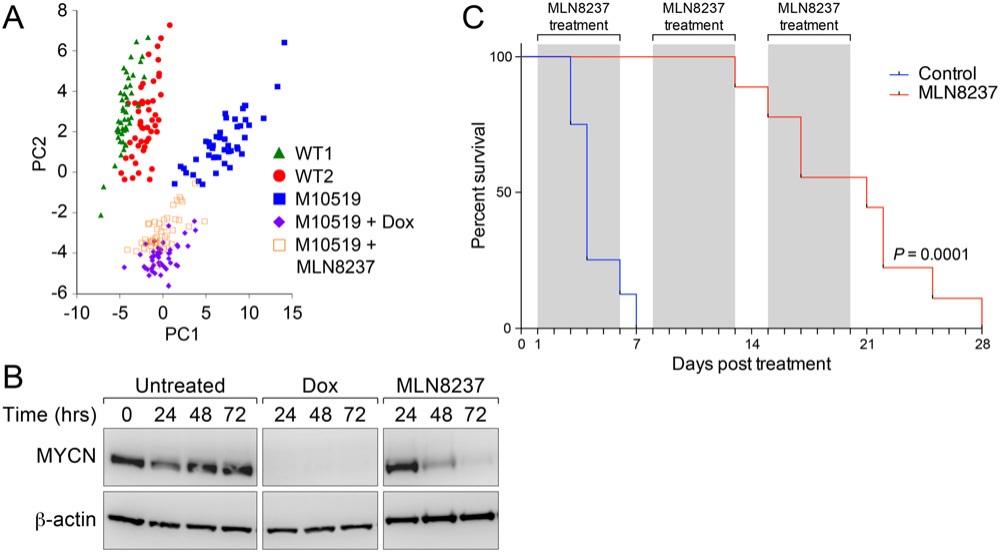
Effect of MLN8237 on GTML spheres and mice. (**A**) PC projections of 96 cells from WT1, WT2, M10519 cells treated with and without dox, and M10519 cells with 100nM MLN8237. PC1 and PC2 captured 21% and 13% of variances in the dataset. (**B**) MYCN stability in MLN8237-treated M10519 cells. Cells were incubated with dox or MLN8237, and cell extracts were analyzed by western blotting. (**C**) Kaplan-Meier curves for overall survival of mice having orthotopic tumors treated with (red) or without MLN8237 (blue). 250 cells were transplanted in to the cerebellum of FBVN mice, and once the tumor burden reached at a level of 1x10^9^ photon/s, mice were daily treated twice with (n = 8) or without (n = 9) MLN8237 (daily oral dosing at 60mg/kg). Mice were treated with MLN8237 for 5 days and then untreated for 2 days, and then the repeat this schedule twice more.

Single cell expression studies suggested that MLN8237 reduces the probability (Fig. 2C) and rate (Fig. 2B) of expression of CD133 similar to dox, suggesting that both effects may similarly diminish the tumor-propagating potential of GTML cells. To evaluate the global impact of MLN8237 on the transcription profile of M10519 GTML line, we conducted principal component analysis (PCA) with single cell expression data. Single cell expression data obtained from wild type neural cells from midbrain (WT1) and from cerebellum (WT2), untreated M10519 cells, compared to cells in which MYCN is targeted either genetically (with dox) or with MLN8237 revealed that cells segregated into two or more subgroups discriminated on the PC1/PC2 axis. On the PCA projection, WT clusters (WT1 and WT2 cells) were located in close proximity, but distanced from the (untreated) M10519 cell cluster. M10519 cells form a distinguished cluster along the PC1 axis, as well as dox- and MLN8237-treated cells formed partially overlapping clusters (Fig. 5A). This profile correlated with the result obtained from a hierarchical clustering analysis. Clusters derived from M10519 cells can be divided into three sub-groups: untreated M10519 cells only, dox-treated M10519 cells only, and cells treated with dox or MLN8237 (S10 Fig.). The first cluster is located away from the second and third clusters, which are more similar to each other. The relatively close distance between M10519 cells+dox and M10519 cells+MLN8237 in two clustering analyses suggested MLN8237 affects regulatory pathways regulated by MYCN. Similar to dox-treated cells, known MYCN targets including E2F1 and E2F2, and neural stem cell markers including CD133, Musashi and Otx2 were downregulated in MLN8237-treated M10519 cells (Fig. 2B, 2C and S5 Fig.). Emergence of these groups correlated with degradation of MYCN as determined by immunoblot analysis (Fig. 5B).

Finally, the effect of MLN8237 on MYCN pathways translated to survival extension in mice bearing well-established tumors. In a survival study, we treated tumors continuously either with vehicle or MLN8237. Mice in the untreated cohort developed lethal tumors within a week, whereas mice in the treatment arm displayed significantly longer survival (Log-rank test, *P* < 0.0001) (Fig. 5C). When we treated mice for 12 days with MLN8237, we observed again longer survival compared to control (Log-rank test, *P* = 0.01) (S11 Fig.). Correlating with these results, immunohistochemistry suggests that treatment of orthotopically-implanted tumors with MLN8237 for 24 hours resulted in modest and dramatic reduction of MYCN and Ki67 levels, respectively (data not shown). These results correlated with our recent observation that MLN8237 inhibited tumor growth and prolonged survival in medulloblastoma-bearing GTML mice, which carry *Trp53* mutation [3].

## Discussion

Here we show that GTML neurospheres represent a powerful tool for analysis of the molecular mechanisms of MYCN-dependent transformation. They are also an efficient platform for *in vitro* and *in vivo* pre-clinical evaluation of small molecule therapeutics for MYCN-driven MB. GTML transgenic mice represent the only native model of MYC oncoprotein family-driven spontaneous medulloblastoma in which genetically manipulable tumors arise in immunocompetent mice with an intact blood-brain-barrier. In this context, it is important that MYCN-driven GTML neurospheres retain the properties of primary tumors when sygeneically implanted *in vivo*. Other recent research into the genetic interaction between MYC or MYCN and p53 has utilized non-natively arising orthotopic tumor implantation models in which either cerebellar granule precursor cells or cerebellar stem cells (CD133+Lin-) were exogenously transduced with either MYC or MYCN and were re-implanted orthotopically into mice [47,48]. In the former publication, transduction of MYCN into cGNP yielded SHH medulloblastoma, and in the latter, coexpression of MYCN with dominant negatively mutated p53 produced large-cell anaplastic medulloblastoma of multiple genetic subgroups. In common with these models, expression microarray analysis has revealed that both the GTML and GTML/*Trp53* ^KI/KI^ models represent Group3 disease [3,12,49]. Although MYCN amplifications are less common than MYC in this subgroup, they are observed at an appreciable frequency, with relevance in all non-WNT subgroups [6–10].

The finding that aggressive MB with features typical of non-SHH human MB is generated by expansion of a highly proliferative and MYCN-dependent tumor-propagating cell that can be maintained in culture and orthotopically transplanted is potentially important for the clinical treatment of aggressive MB. The majority of these tumors are not defined by discrete genetic changes or alterations in pathway signaling that are druggable. Our finding that growth of tumor-propagating cells in this system are MYCN-dependent, and to some extent can be forced to differentiate by targeted inhibition of MYCN, resulting in regression of tumors highlights the therapeutic potential of MYCN-targeted treatment approaches for patients with aggressive MB. It should also be noted, however, that MYCN withdrawal only caused the cell cycle arrest in GTML spheres and the temporal remission of orthotropic tumors in a tissue environment; we observed that prolonged (> 2 weeks) treatment of GTML mice with dox occasionally resulted in the emergence of dox-resistant tumors (data not shown). Phenotypically, the relapsed tumors resembled the original orthotopic tumors, expressing MYCN, Ki67, but not GFAP in the presence of dox (data not shown). These results suggest that there appears to be small number of cancer cells, in which MYCN expression is not controlled by rTA only. Alternatively, dox may not be efficiently delivered to a part of tissues, from which the outgrowth of new tumors may occur. Regardless of the mechanism, these results clearly suggest that targeting MYCN with a single drug may only result in the short-term remission, and thus a combinatorial treatment with other drugs may be necessarily to eradicate cancer cells.

While GTML neurospheres appear to be a powerful tool to study the nature of cancer-propagating cells, we found apparent inconsistencies in differentiation potential between cells cultured as neurospheres or implanted orthotopically into the cerebellum. GTML cells retain sphere morphology reminiscent of undifferentiated cells in culture in the presence of differentiation inducers following MYCN withdrawal. In orthotopic tumors, we detected evidence of enhanced staining for markers associated with neuronal and glial differentiation. This discrepancy can be explained by the fact that the latter was examined in a tissue environment, while the former was examined in culture without any biological niche. Since we found that MYCN withdrawal in GTML neurospheres results in the decrease of Nestin after 2 days, certain levels of differentiation may be triggered in dox-treated GTML cells. Culturing GTML cells in a microenvironment that is permissive to differentiation, for example, with astrocytes [50] may help establishing a more advanced pre-clinical system that recapitulates the nature of tumor cells in a tissues environment.

## Supporting Information

S1 Fig Fine-tuning of tumor growth in the GTML system. (**A**) Simultaneous tissue specific expression of human MYCN and luciferase is driven by TRE-mediated expression in a target mouse (TRE-MYCN-Luc, TML). Mating of single-transgenic TML mice with those expressing the Tetracycline transactivator (tTA) under the control of the Glutamate transporter (GLT1) promoter (GLT1-tTA) provides CNS specificity confined to the hindbrain. Dox (Dox)-mediated inhibition of TRE activation allows robust genetic control of MYCN and luciferase (Luc) expression. (**B**) Monitoring tumor growth through bioluminescence. Rapid tumor growth from days 81 to 99 is shown (**C**) M10519 cells were cultured and then incubated with luciferin, and then the luciferase signals were measured. The bioluminescence signal was correlated with the number of spheres or cells. Error bars, ±SD. (**D**) Effect of growth factors (GF) on three GTML lines (M0983, M14942, and M10519). Spheres were cultured with or without 20ng/ml of bFGF and EGF and cell numbers were counted. Error bars, ±SD. (**E**) Re-entry to growth after removal of dox. Three GTML lines (M0983, M21446, and M10519) were treated with dox for 7 days, and then cells were cultured without dox. Error bars, ±SD. (**F**) Stability of MYCN and c-Myc proteins upon dox treatment. Cell extracts from M21446 GTML cells were examined by western analyses. Spheres were cultured in the presence or absence of dox (1 or 3μg/ml) and harvested at 6 hours.
(TIF)

S2 Fig Growth and differentiation characteristics of GTML spheres. (**A**) Effect of MYCN withdrawal and differentiation inducers on M10519 GTML cells. M10519 GTML spheres were cultured in neurobasal media with growth factors and either vehicle, dox (1μg/ml) or pro-differentiation containing serum and retinoic acid (Diff. Media) as indicated and sphere formation and bioluminescence signals were monitored. Bar, 100μm. (**B**) Effect of serum and dox on three GTML lines (M14942, M0982, and M10519) and wild type cells from the cerebellum. Spheres were cultured for 8 days in neurobasal media with growth factors and either vehicle, dox (1μg/ml), serum, or pro-differentiation containing serum and retinoic acid (Diff. Media) as indicated. Bar, 100μm.
(TIF)

S3 Fig Protein marker expression profiles in GTML spheres. (**A**) Impact of MYCN withdrawal and differentiation inducers on marker expression in M10519 GTML cells. M10519 GTML spheres were cultured in neurobasal media with growth factors and either vehicle, dox (1μg/ml) or pro-differentiation containing serum and retinoic acid (Diff. Media) as indicated. (**B**) M10519 GTML spheres were treated with vehicle or dox for 7 days and expression of Cleaved Caspase 3 and MYCN analyzed by immunofluorescence. Nuclei were counterstained with DAPI. Bar, 50μm.
(TIF)

S4 Fig Limiting-dilution sphere assay using M10519 cells. Serial dilutions (100, 10 and 1 cells per well) GTML cells were cultured in neurobasal media with B27 and growth factors. The numbers of wells containing spheres were counted.
(TIF)

S5 Fig Expression analysis of M10519 GTML cells. Heat map showing expression levels (Cq values) of 96 genes. Indicated are wild-type cells from midbrain (WT1) or cerebellum (WT2), untreated M10519 spheres (M10519), M10519 spheres treated with dox for 24 hours (+Dox), or M10519 spheres treated with MLN8237 for 24 hours (+MLN8237). Mean expression values obtained from 96 single cells for each condition are shown.
(TIF)

S6 Fig Single cell Expression analysis of M10519 GTML cells. Heat map showing expression levels (Cq values) of 96 genes from single cells (n = 96 cells for each condition). Indicated are wild-type cells from midbrain (WT1) or cerebellum (WT2), untreated M10519 spheres (M10519), M10519 spheres treated with dox for 24 hours (M10519+Dox), or M10519 spheres treated with MLN8237 for 24 hours (M10519+MLN8237).
(TIF)

S7 Fig Characterization of GTML spheres by orthotopic implantation. (**A**) Serial dilutions of M10519 GTML cells (passage 10–27) were implanted into the cerebellum of immunocompetent (FVB/N) mice: n = 10 (for 1000, 5000, 1000, 250, and 100 cells); n = 9 (for 50 and 25 cells); n = 10 for tumor cells implanted without *in vitro* expansion. Tumor incidence was evaluated by monitoring bioluminescence twice per week. (**B**) Kaplan-Meier curve showing overall survival of mice implanted with M14942 (blue, passage 11, n = 5), and M10519 (red, passage 10, n = 5) cells. 250 cells were implanted orthotopically per site.
(TIF)

S8 Fig Tumor-propagating potential of FACS-sorted CD15+ cells. (**A**) Sorting of CD15+ and CD15- populations from M21446 GTML cells by FACS. (**B, C**) Kaplan-Meier curves for overall survival of mice implanted with CD15+ or CD15- cells from (**B**) M21446 (passage 20) and (**C**) M0983 (passage 10) cells. 10 cells were implanted into the cerebellum per mouse (n = 5 for each). (**D**) Sphere assays using FACS-sorted CD15+ and CD15- cells (M10519 cells, passage 18). 50 cells per well were plated onto a 24-well plate containing neurobasal media in the presence of growth factors and collagen (1mg/ml) and then cultured for four weeks.
(TIF)

S9 Fig Tumor-propagating potential of FACS-sorted CD133+ cells. (**A**) Sorting of CD133+ and CD133- populations from M10519 GTML cells (passage 23) by FACS. Cells were incubated with control IgG1 or anti-CD133 conjugated with PE prior to sorting. (**B**) Kaplan-Meier curve showing overall survival of mice implanted with CD133+ or CD133- cells. 10 cells were implanted into the cerebellum per mouse (n = 5 for each). (**C**) 3D neurosphere assays using FACS-sorted CD133+ and CD133- cells (M10519, passage 23). 50 cells per well were plated onto a 24-well plate containing neurobasal media in the presence of growth factors and collagen (1mg/ml) and then cultured for four weeks.
(TIF)

S10 Fig Hierarchical clustering analysis of GTML following treatment with dox or MLN8237. “M10519” cluster (black), “M10519+dox” cluster (green), and “M10519+MLN8237 plus M10519+Dox cluster” (blue) are shown.
(TIF)

S11 Fig Effect of MLN8237 on GTML mice. Kaplan-Meier curves for overall survival of mice bearing orthotopic tumors treated with MLN8237 (red) or vehicle (blue). 250 cells (M10519 cells, passage 16) were transplanted into the cerebellum of FBVN mice and treatment initiated when tumor-associated bioluminescence signal reached 1x10^9^ photon/s. Mice were treated by oral gavage (30mg/kg twice daily) with MLN8237 (red, n = 4) or vehicle (blue, n = 4). Mice were treated with MLN8237 or vehicle for a period 12 days as indicated by grey shading.
(TIF)

S1 Table List of 96 genes with primer sequences used in BioMark qPCR. (XLSX)

S2 Table Characteristics of GTML tumor-derived neurosphere cell lines. (DOCX)

S3 Table BioMark qPCR expression data. (XLSX)
