# Supplementary figures and images for: Molecular and In Vivo Characterization of Cancer-Propagating Cells Derived from MYCN-Dependent Medulloblastoma
Source: PLoS One. 2015 Mar 18;10(3):e0119834. doi: 10.1371/journal.pone.0119834 (PMC4365014; doi:10.1371/journal.pone.0119834)

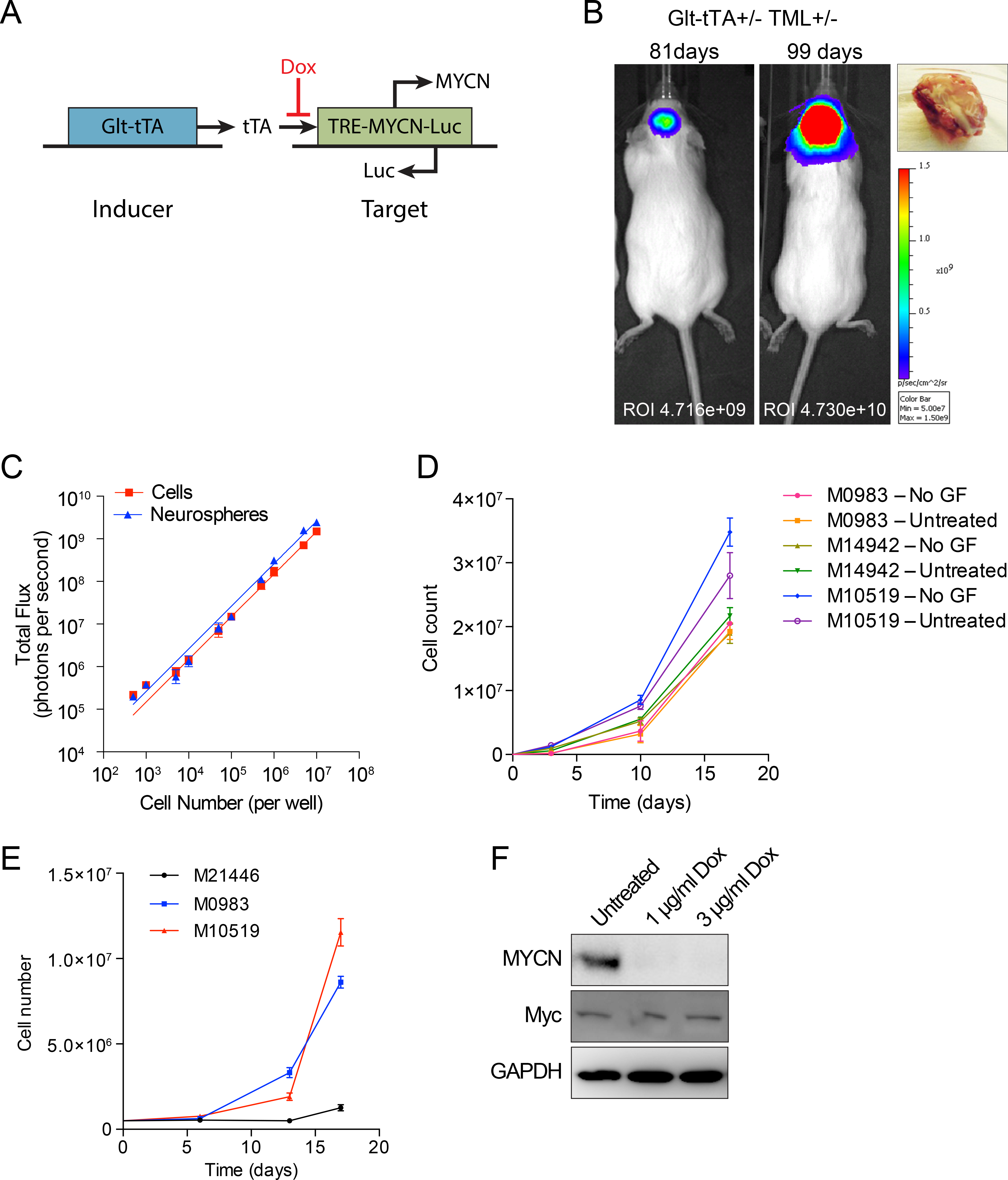

Supplement: S1 Fig — (A) Simultaneous tissue specific expression of human MYCN and luciferase is driven by TRE-mediated expression in a target mouse (TRE-MYCN-Luc, TML). Mating of single-transgenic TML mice with those expressing the Tetracycline transactivator (tTA) under the control of the Glutamate transporter (GLT1) promoter (GLT1-tTA) provides CNS specificity confined to the hindbrain. Dox (Dox)-mediated inhibition of TRE activation allows robust genetic control of MYCN and luciferase (Luc) expression. (B) Monitoring tumor growth through bioluminescence. Rapid tumor growth from days 81 to 99 is shown (C) M10519 cells were cultured and then incubated with luciferin, and then the luciferase signals were measured. The bioluminescence signal was correlated with the number of spheres or cells. Error bars, ±SD. (D) Effect of growth factors (GF) on three GTML lines (M0983, M14942, and M10519). Spheres were cultured with or without 20ng/ml of bFGF and EGF and cell numbers were counted. Error bars, ±SD. (E) Re-entry to growth after removal of dox. Three GTML lines (M0983, M21446, and M10519) were treated with dox for 7 days, and then cells were cultured without dox. Error bars, ±SD. (F) Stability of MYCN and c-Myc proteins upon dox treatment. Cell extracts from M21446 GTML cells were examined by western analyses. Spheres were cultured in the presence or absence of dox (1 or 3μg/ml) and harvested at 6 hours. (TIF) [file pone.0119834.s001.tif]

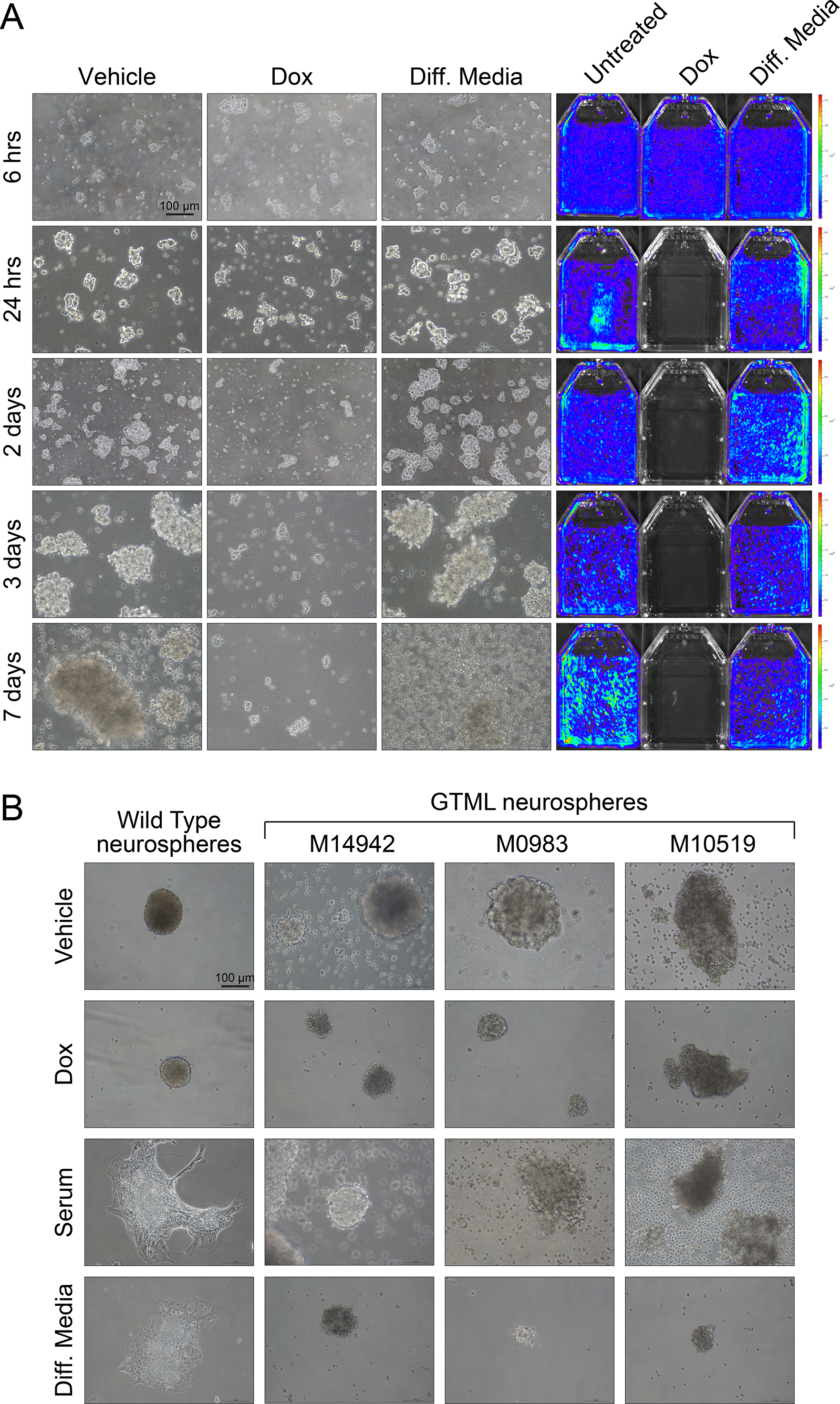

Supplement: S2 Fig — (A) Effect of MYCN withdrawal and differentiation inducers on M10519 GTML cells. M10519 GTML spheres were cultured in neurobasal media with growth factors and either vehicle, dox (1μg/ml) or pro-differentiation containing serum and retinoic acid (Diff. Media) as indicated and sphere formation and bioluminescence signals were monitored. Bar, 100μm. (B) Effect of serum and dox on three GTML lines (M14942, M0982, and M10519) and wild type cells from the cerebellum. Spheres were cultured for 8 days in neurobasal media with growth factors and either vehicle, dox (1μg/ml), serum, or pro-differentiation containing serum and retinoic acid (Diff. Media) as indicated. Bar, 100μm. (TIF) [file pone.0119834.s002.tif]

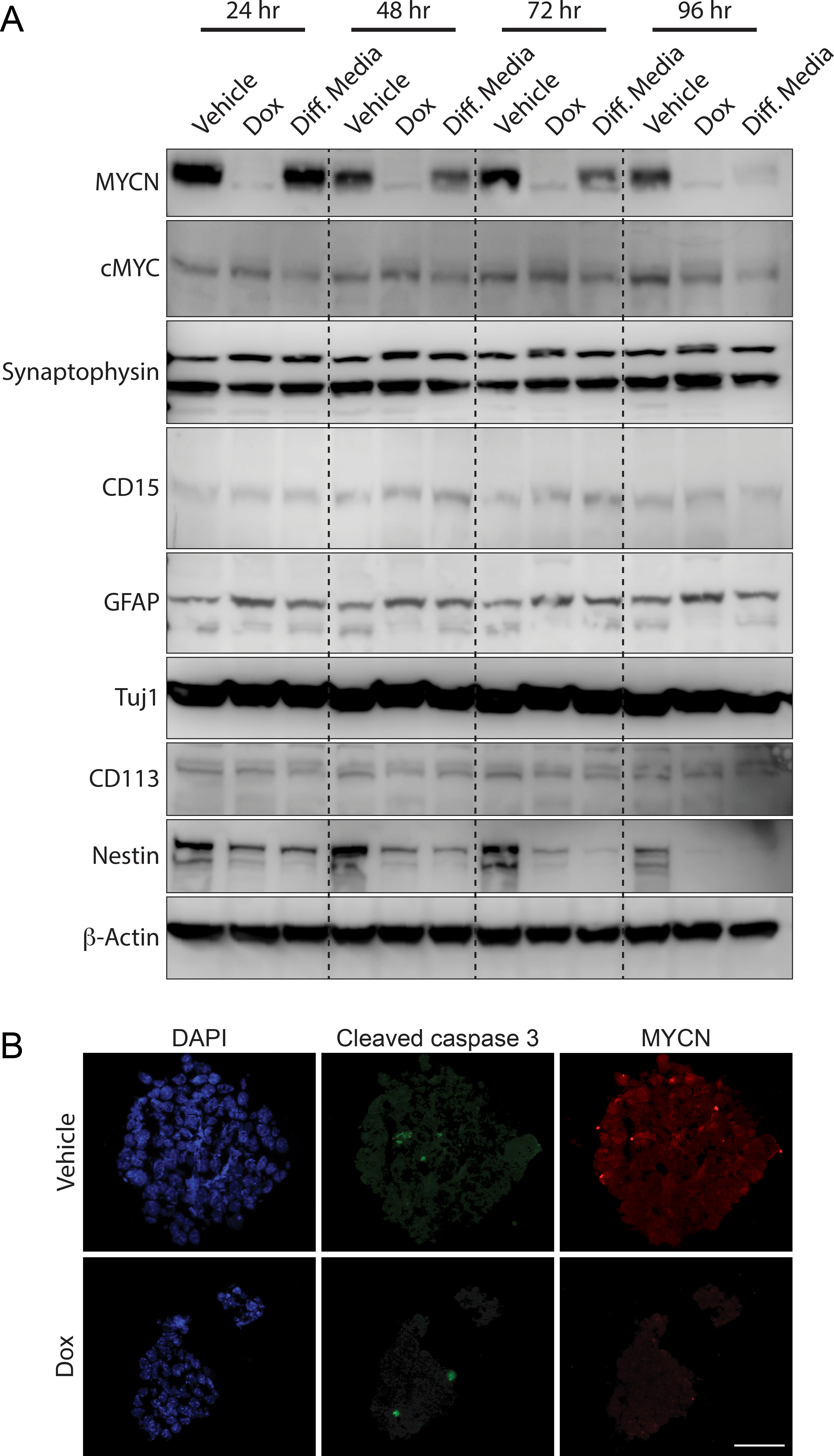

Supplement: S3 Fig — (A) Impact of MYCN withdrawal and differentiation inducers on marker expression in M10519 GTML cells. M10519 GTML spheres were cultured in neurobasal media with growth factors and either vehicle, dox (1μg/ml) or pro-differentiation containing serum and retinoic acid (Diff. Media) as indicated. (B) M10519 GTML spheres were treated with vehicle or dox for 7 days and expression of Cleaved Caspase 3 and MYCN analyzed by immunofluorescence. Nuclei were counterstained with DAPI. Bar, 50μm. (TIF) [file pone.0119834.s003.tif]

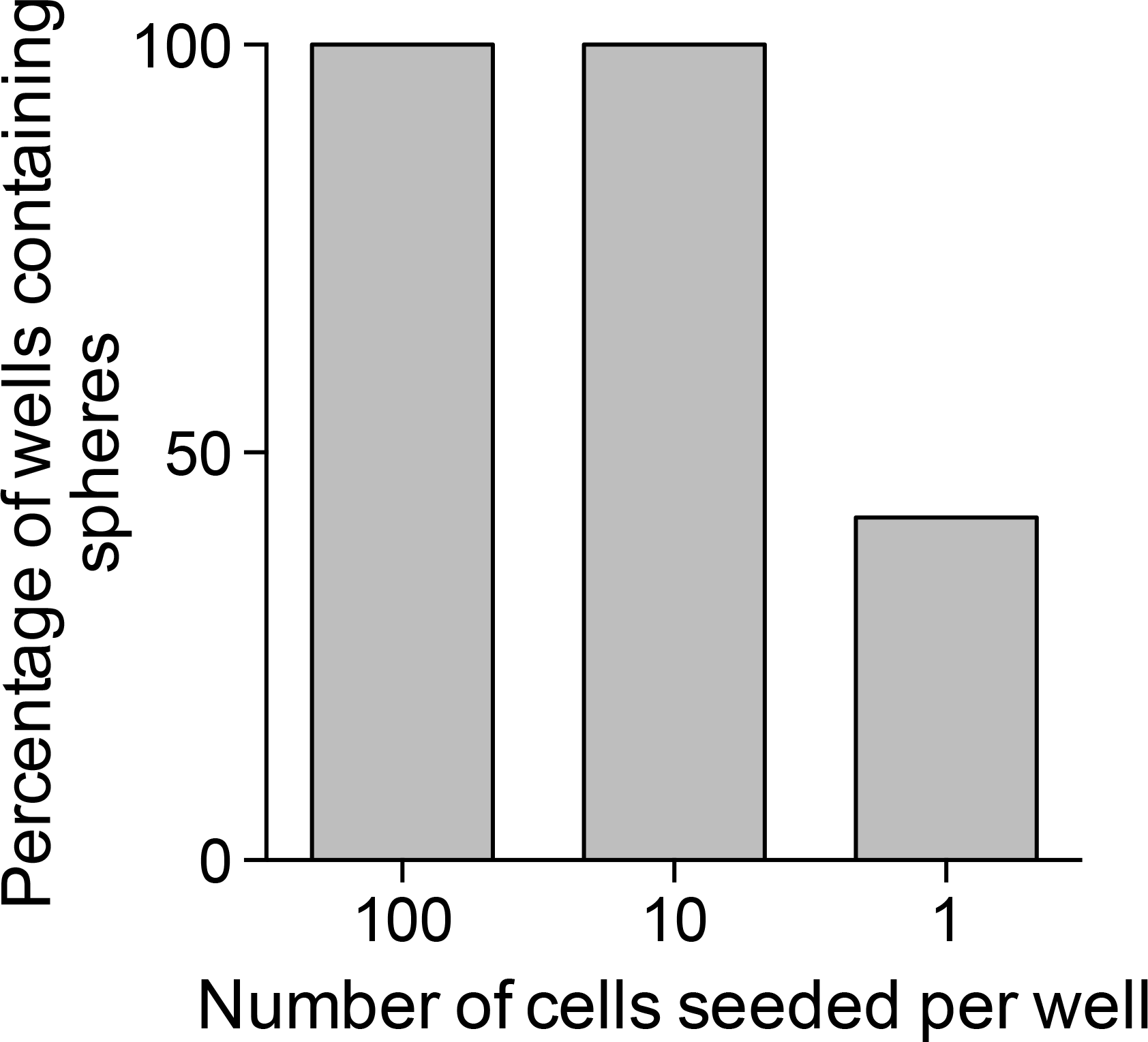

Supplement: S4 Fig — Serial dilutions (100, 10 and 1 cells per well) GTML cells were cultured in neurobasal media with B27 and growth factors. The numbers of wells containing spheres were counted. (TIF) [file pone.0119834.s004.tif]

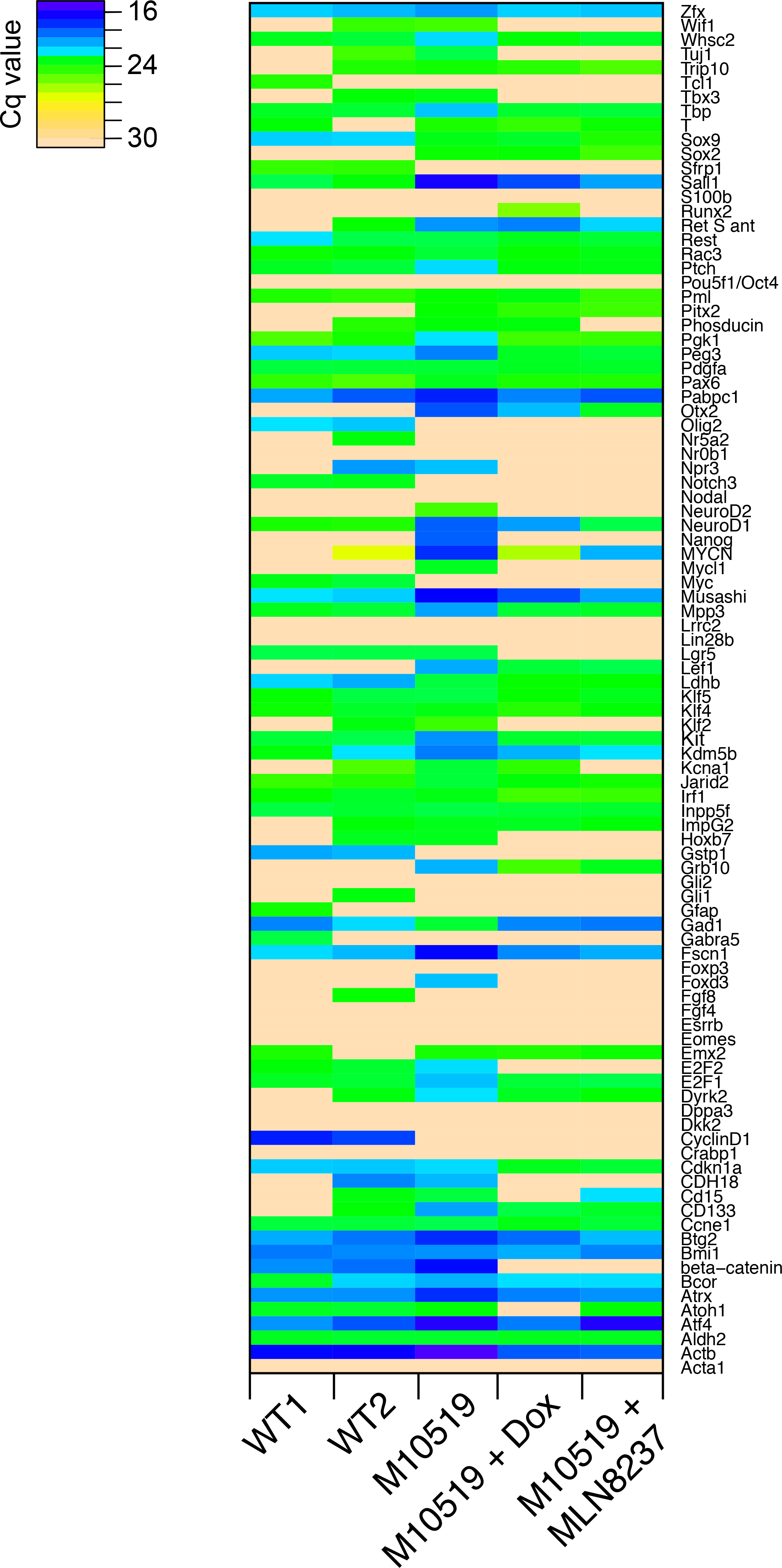

Supplement: S5 Fig — Heat map showing expression levels (Cq values) of 96 genes. Indicated are wild-type cells from midbrain (WT1) or cerebellum (WT2), untreated M10519 spheres (M10519), M10519 spheres treated with dox for 24 hours (+Dox), or M10519 spheres treated with MLN8237 for 24 hours (+MLN8237). Mean expression values obtained from 96 single cells for each condition are shown. (TIF) [file pone.0119834.s005.tif]

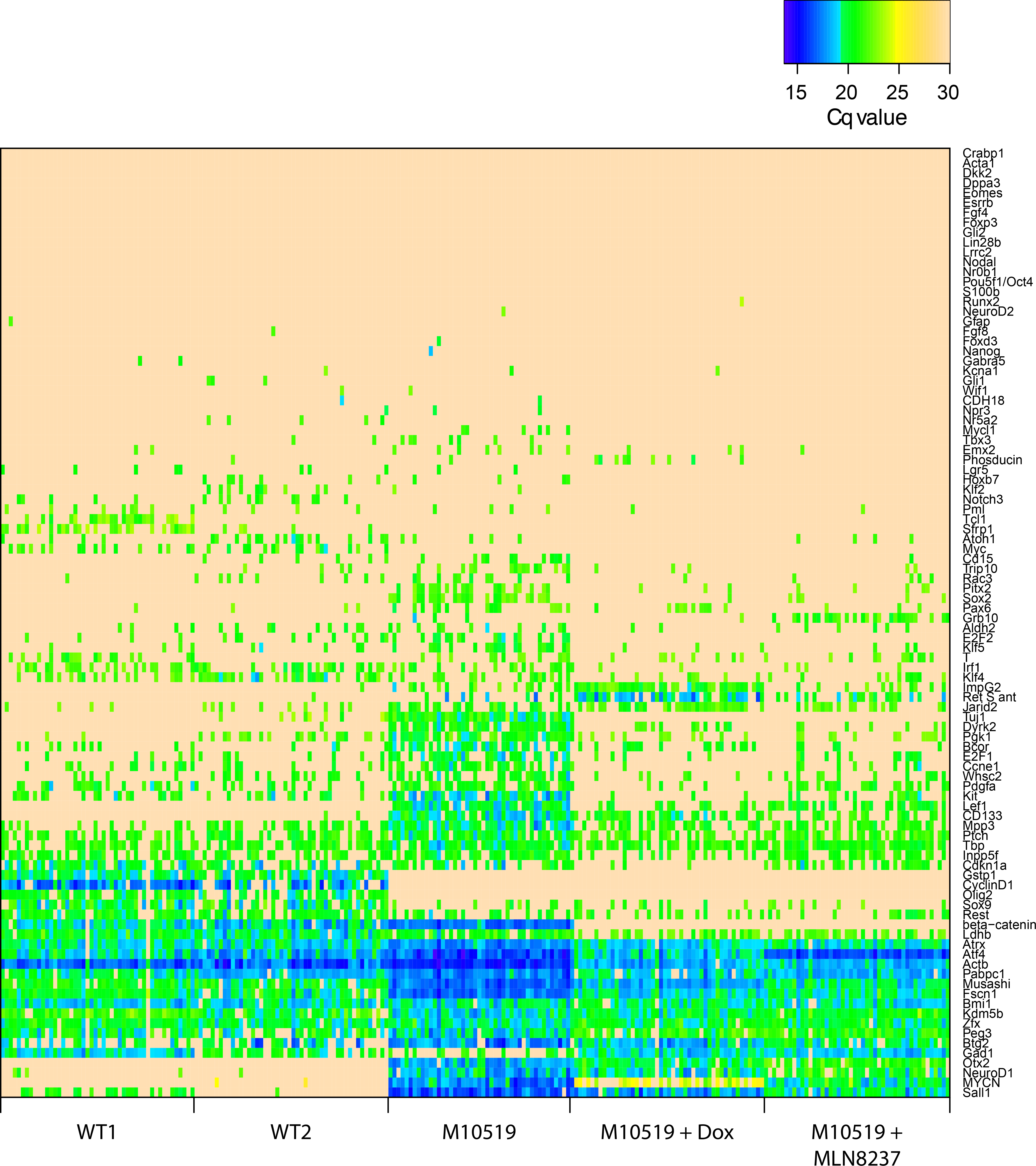

Supplement: S6 Fig — Heat map showing expression levels (Cq values) of 96 genes from single cells (n = 96 cells for each condition). Indicated are wild-type cells from midbrain (WT1) or cerebellum (WT2), untreated M10519 spheres (M10519), M10519 spheres treated with dox for 24 hours (M10519+Dox), or M10519 spheres treated with MLN8237 for 24 hours (M10519+MLN8237). (TIF) [file pone.0119834.s006.tif]

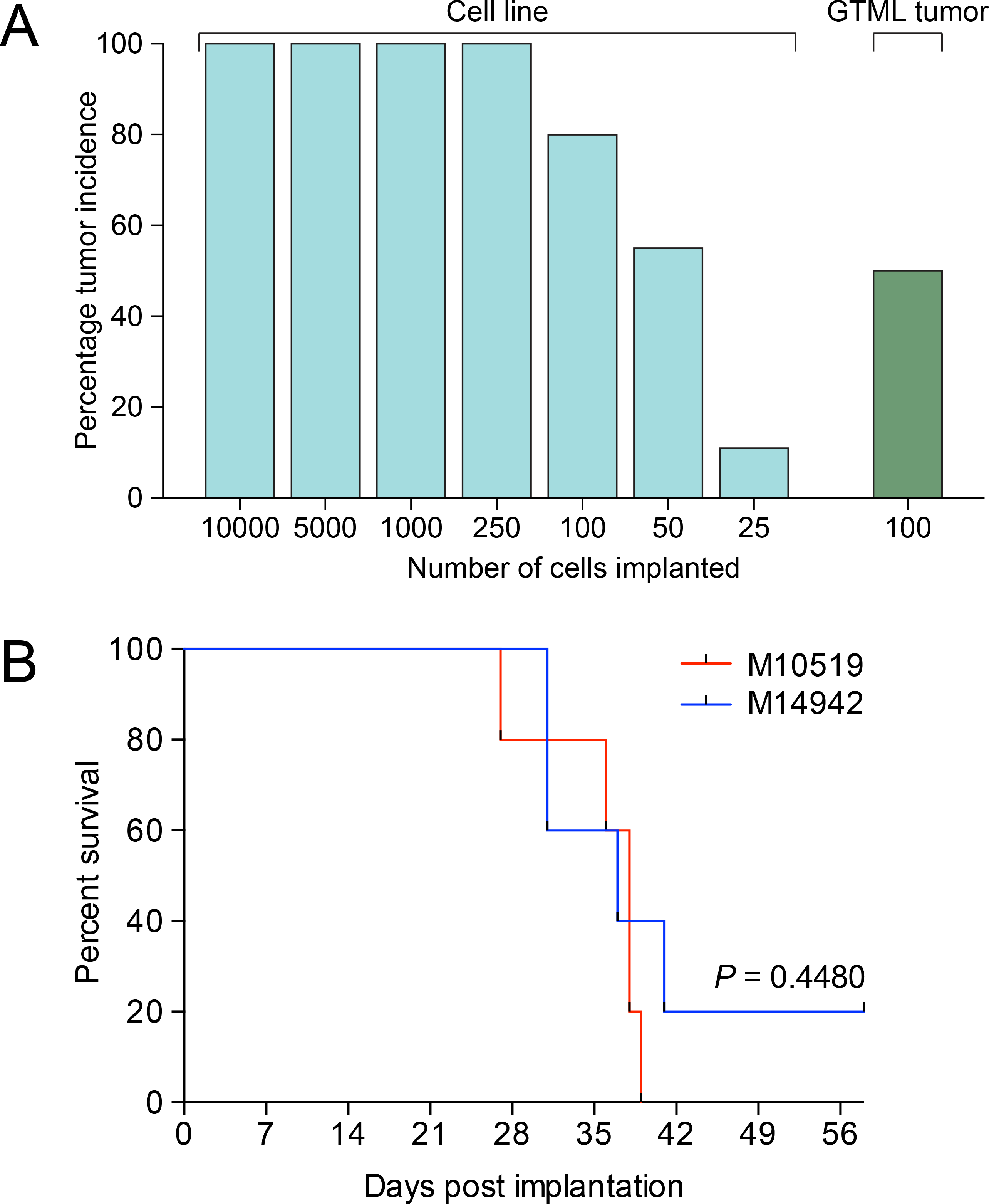

Supplement: S7 Fig — (A) Serial dilutions of M10519 GTML cells (passage 10–27) were implanted into the cerebellum of immunocompetent (FVB/N) mice: n = 10 (for 1000, 5000, 1000, 250, and 100 cells); n = 9 (for 50 and 25 cells); n = 10 for tumor cells implanted without in vitro expansion. Tumor incidence was evaluated by monitoring bioluminescence twice per week. (B) Kaplan-Meier curve showing overall survival of mice implanted with M14942 (blue, passage 11, n = 5), and M10519 (red, passage 10, n = 5) cells. 250 cells were implanted orthotopically per site. (TIF) [file pone.0119834.s007.tif]

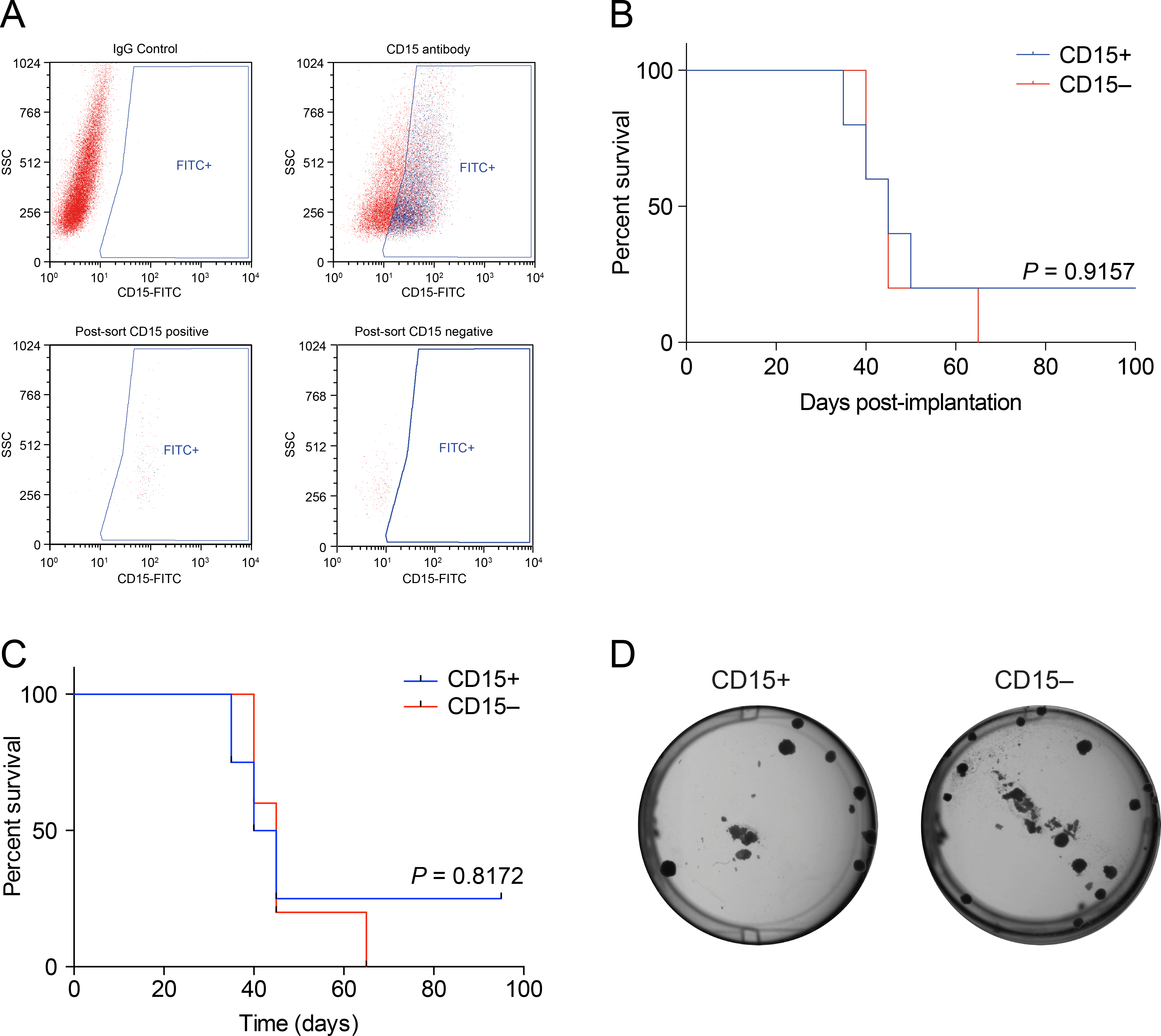

Supplement: S8 Fig — (A) Sorting of CD15+ and CD15- populations from M21446 GTML cells by FACS. (B, C) Kaplan-Meier curves for overall survival of mice implanted with CD15+ or CD15- cells from (B) M21446 (passage 20) and (C) M0983 (passage 10) cells. 10 cells were implanted into the cerebellum per mouse (n = 5 for each). (D) Sphere assays using FACS-sorted CD15+ and CD15- cells (M10519 cells, passage 18). 50 cells per well were plated onto a 24-well plate containing neurobasal media in the presence of growth factors and collagen (1mg/ml) and then cultured for four weeks. (TIF) [file pone.0119834.s008.tif]

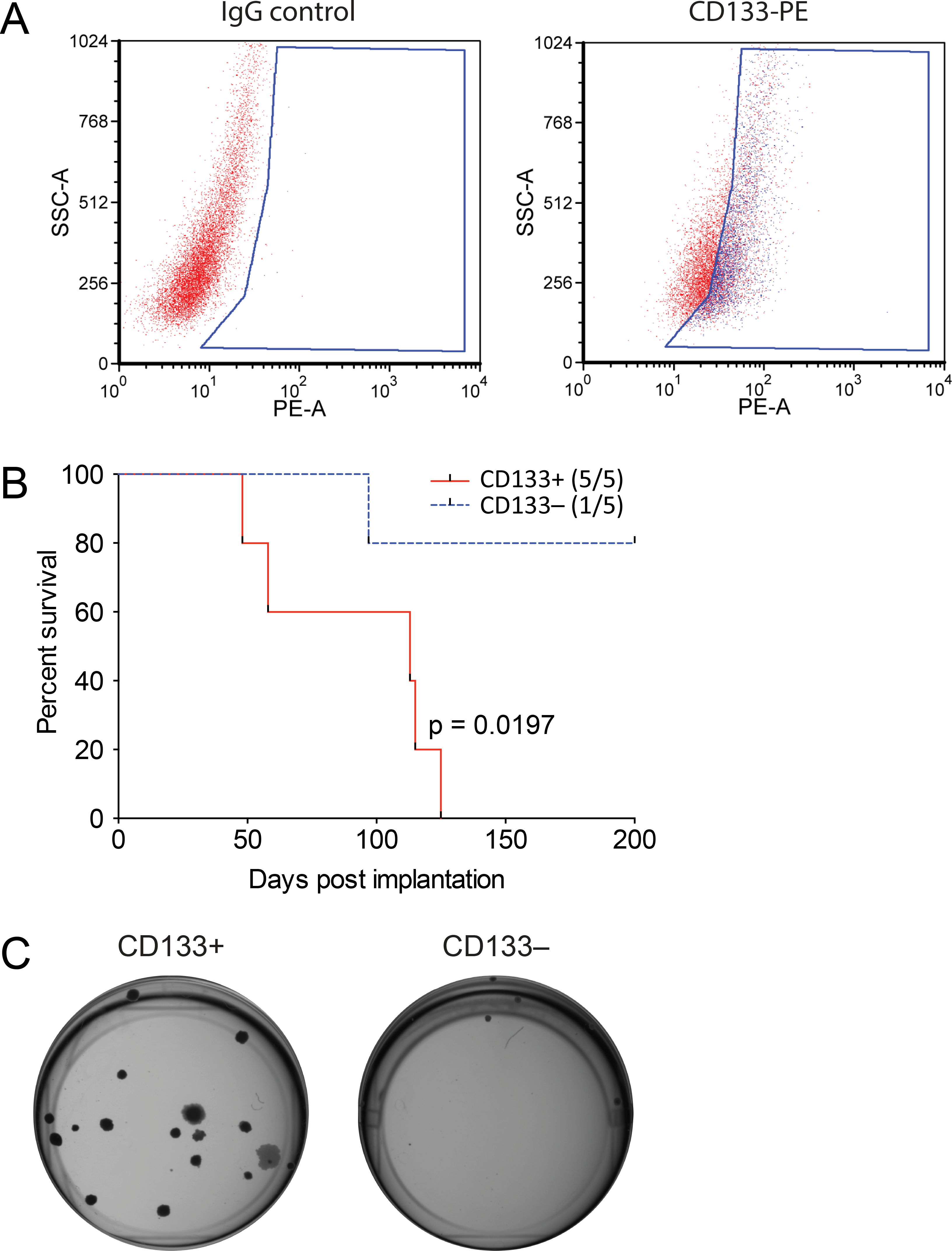

Supplement: S9 Fig — (A) Sorting of CD133+ and CD133- populations from M10519 GTML cells (passage 23) by FACS. Cells were incubated with control IgG1 or anti-CD133 conjugated with PE prior to sorting. (B) Kaplan-Meier curve showing overall survival of mice implanted with CD133+ or CD133- cells. 10 cells were implanted into the cerebellum per mouse (n = 5 for each). (C) 3D neurosphere assays using FACS-sorted CD133+ and CD133- cells (M10519, passage 23). 50 cells per well were plated onto a 24-well plate containing neurobasal media in the presence of growth factors and collagen (1mg/ml) and then cultured for four weeks. (TIF) [file pone.0119834.s009.tif]

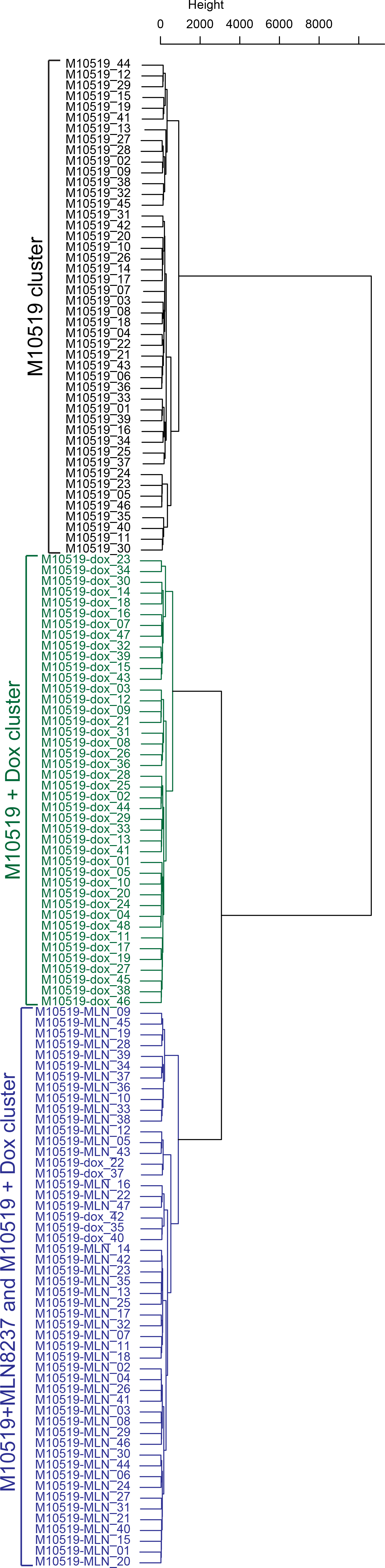

Supplement: S10 Fig — “M10519” cluster (black), “M10519+dox” cluster (green), and “M10519+MLN8237 plus M10519+Dox cluster” (blue) are shown. (TIF) [file pone.0119834.s010.tif]

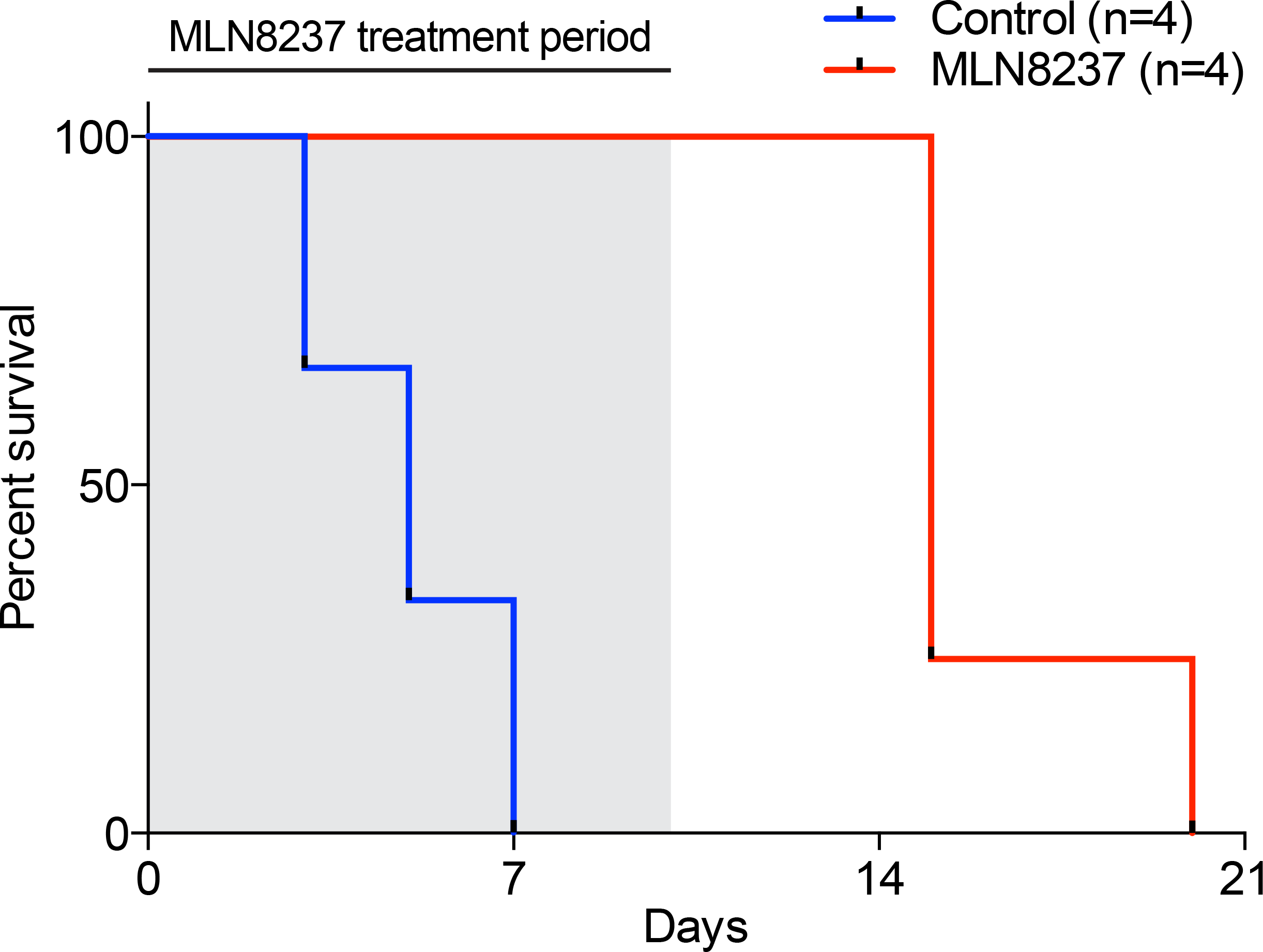

Supplement: S11 Fig — Kaplan-Meier curves for overall survival of mice bearing orthotopic tumors treated with MLN8237 (red) or vehicle (blue). 250 cells (M10519 cells, passage 16) were transplanted into the cerebellum of FBVN mice and treatment initiated when tumor-associated bioluminescence signal reached 1x10^9^ photon/s. Mice were treated by oral gavage (30mg/kg twice daily) with MLN8237 (red, n = 4) or vehicle (blue, n = 4). Mice were treated with MLN8237 or vehicle for a period 12 days as indicated by grey shading. (TIF) [file pone.0119834.s011.tif]
