# Supplementary material for: Molecular and In Vivo Characterization of Cancer-Propagating Cells Derived from MYCN-Dependent Medulloblastoma
Source: PLoS One. 2015 Mar 18;10(3):e0119834. doi: 10.1371/journal.pone.0119834 (PMC4365014; doi:10.1371/journal.pone.0119834)
Supplement: S2 Table — (DOCX) [file pone.0119834.s013.docx]

| **S2 Table. Characteristics of GTML tumor-derived neurosphere cell lines** | | | | | |
| --- | --- | --- | --- | --- | --- |
| **Cell line ID** | **Tissue of origin** | **Age of mice** | **Appearance in culture** | **Histology of orthotopic tumor** | **Doubling time** |
| **M10519** | GTML cerebellum | 121 | Large irregular spheres | Large Cell Anaplastic | 24 hours |
| **M0983** | GTML cerebellum | 112 | Large irregular spheres | Large Cell Anaplastic | 24 hours |
| **M21446** | GTML cerebellum | 169 | Large regular spheres | ND | 24 hours |
| **M6257** | GTML cerebellum | 93 | Large regular spheres | ND | ND |
| **M0983** | GTML cerebellum | 169 | Large irregular spheres | Large Cell Anaplastic | 24 hours |
| **M4058** | GTML cerebellum | 196 | Mainly adherent with small spheres | Large Cell Anaplastic | ND |
| ND: not determined | | | | | |
